# Deep proteomics reveals incorporation of unedited proteins into mitochondrial protein complexes in Arabidopsis

**DOI:** 10.1093/plphys/kiad655

**Published:** 2023-12-07

**Authors:** Nils Rugen, Michael Senkler, Hans-Peter Braun

**Affiliations:** Institute of Plant Genetics, Leibniz Universität Hannover, Herrenhäuser Str. 2, 30419 Hannover, Germany; Institute of Plant Genetics, Leibniz Universität Hannover, Herrenhäuser Str. 2, 30419 Hannover, Germany; Institute of Plant Genetics, Leibniz Universität Hannover, Herrenhäuser Str. 2, 30419 Hannover, Germany

## Abstract

The mitochondrial proteome consists of numerous types of proteins which either are encoded and synthesized in the mitochondria, or encoded in the cell nucleus, synthesized in the cytoplasm and imported into the mitochondria. Their synthesis in the mitochondria, but not in the nucleus, relies on the editing of the primary transcripts of their genes at defined sites. Here, we present an in-depth investigation of the mitochondrial proteome of Arabidopsis (*Arabidopsis thaliana*) and a public online platform for the exploration of the data. For the analysis of our shotgun proteomic data, an Arabidopsis sequence database was created comprising all available protein sequences from the TAIR10 and Araport11 databases, supplemented with sequences of proteins translated from edited and nonedited transcripts of mitochondria. Amino acid sequences derived from partially edited transcripts were also added to analyze proteins encoded by the mitochondrial genome. Proteins were digested in parallel with six different endoproteases to obtain maximum proteome coverage. The resulting peptide fractions were finally analyzed using liquid chromatography coupled to ion mobility spectrometry and tandem mass spectrometry. We generated a “deep mitochondrial proteome” of 4,692 proteins. 1,339 proteins assigned to mitochondria by the SUBA5 database (https://suba.live) accounted for >80% of the total protein mass of our fractions. The coverage of proteins by identified peptides was particularly high compared to single-protease digests, allowing the exploration of differential splicing and RNA editing events at the protein level. We show that proteins translated from nonedited transcripts can be incorporated into native mitoribosomes and the ATP synthase complex. We present a portal for the use of our data, based on “proteomaps” with directly linked protein data. The portal is available at www.proteomeexplorer.de.

## Teaser

Partially edited mitochondrial transcripts may be translated and the resulting proteins incorporated into native protein complexes.

## Introduction

Plant mitochondria are involved in various processes, most notably cellular respiration and photorespiration (Millar et al. 2011). The different mitochondrial functions are carried out by specific sets of proteins, which together form the plant mitochondrial proteome, the entirety of all proteins present in the mitochondria of plants. Shotgun proteomics is the primary method employed to study mitochondrial proteomes, wherein proteins are fragmented into peptides by trypsin, and the resulting peptides are subsequently identified and quantified via liquid chromatography coupled mass spectrometry (LC–MS) analysis. This approach has been successfully used in numerous studies to characterize the plant mitochondrial proteome composition (Heazlewood et al. 2004; Salvato et al. 2014; Rao et al. 2017) and recently even allowed to define the inventory of a single plant mitochondrion (Fuchs et al. 2020). It has given insights into post-translational modifications (PTMs) (König et al. 2014; Hartl et al. 2017) and permitted to follow the protein turnover in the mitochondria of plants (Huang et al. 2020). It improved our knowledge of protein complex composition and dynamics (Senkler et al. 2017; Takabayashi et al. 2017; Rugen et al. 2021), allowed to follow rearrangements of the respiratory chain in plants lacking complex I (Fromm et al. 2016; Maclean et al. 2018; Senkler et al. 2018) and to define the composition of the plant mitochondrial ribosome (Rugen et al. 2019).

The complexity of the plant mitochondrial proteome is further increased by the fact that mitochondrial transcripts can be edited at specific sites (Small et al. 2020; Knoop 2022). RNA editing is mainly caused by the deamination of cytidine to uridine, which can lead to modifications in the amino acid sequences of the encoded proteins. Since RNA editing at specific sites occurs at different frequencies (Bentolila et al. 2013), partially edited transcripts might also be used for translation. Indeed, such transcripts have been shown to be loaded onto mito-ribosomes (Planchard et al. 2018). Translation of partially edited transcripts could result in protein polymorphisms that may have an impact on protein activity, localization, or protein–protein interactions (Knoop 2022). However, differential RNA editing has not been shown to result in protein heterogeneity. Instead, the editing of mitochondrial RNAs rather is considered a repair process that compensates for defective sequences in mitochondrial genes (Small et al. 2020). This assumption is supported by the absence of proteins corresponding to non- or partially edited transcripts in wild-type plants (Small et al. 2023). However, it has not been clarified whether putative “nonedited proteins” are synthesized but largely degraded or, alternatively, their synthesis is avoided. Transcripts could be checked for complete editing during translation, and editing of partially edited transcripts is co-translationally completed if necessary. Indeed, it is hypothesized that single PPR proteins or assemblies of various editing factors (so-called editosomes) can occupy the mRNA and, thus, pause translation until full editing is achieved. This could also explain the observed increase in the editing state of ribosome-associated transcripts (Planchard et al. 2018; Small et al. 2020). However, so far, protein polymorphisms caused by RNA editing have not been studied systemically on the proteomic level.

Here, we present a study to characterize the mitochondrial proteome of Arabidopsis (*Arabidopsis thaliana*) in considerable depth. We apply a shotgun proteomics strategy based on protein digestion using six different proteases (trypsin, Arg-C, Lys-C, Asp-N, chymotrypsin, and Glu-C). The resulting fractions are analyzed using sensitive liquid chromatography coupled with ion mobility spectrometry and tandem mass spectrometry (LC-IMS-MS/MS). The vast majority of mitochondrially encoded proteins are derived from edited mRNAs, suggesting that complete maturation of transcripts is a prerequisite for the biosynthesis of the corresponding proteins. However, we report exceptions for a subunit of the ATP synthase complex and for subunits of the mitoribosomes. Re-analysis of a recently published complexome profiling dataset revealed that proteins derived from incompletely edited transcripts are incorporated into the respective native protein complexes. Furthermore, we introduce the Proteome Explorer tool, which facilitates the user-friendly exploration of extensive proteome datasets through linking them with interactive “proteomaps” in a web interface. Our deep mitochondrial proteome dataset is available at www.proteomeexplorer.de.

## Results and discussion

### A multiprotease approach for deep analysis of the mitochondrial proteome of *Arabidopsis thaliana*

**Figure 1.**
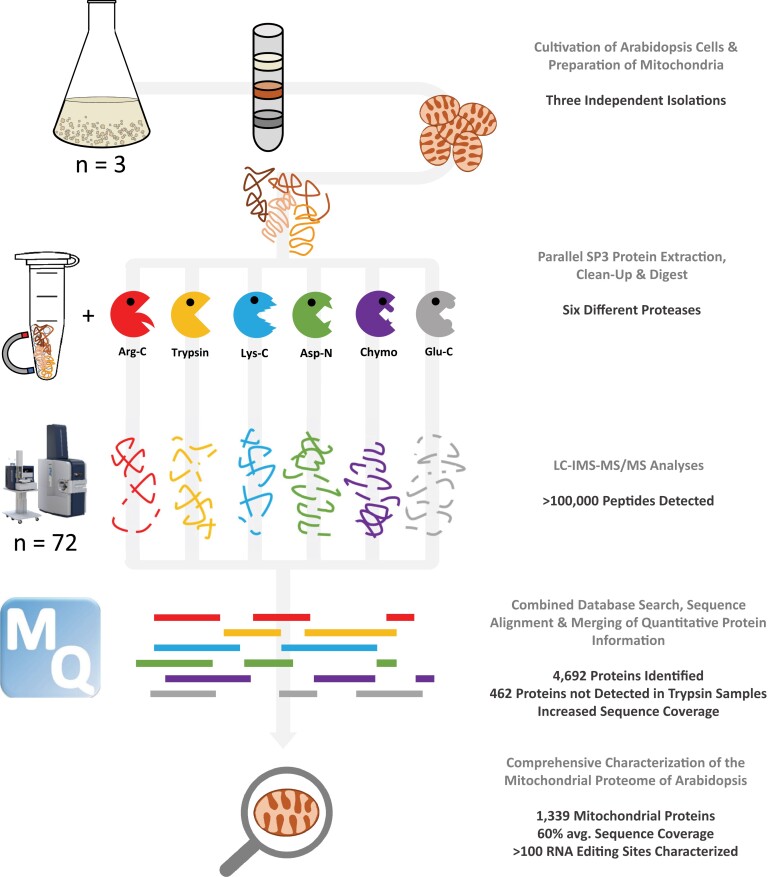
Schematic representation of the experimental workflow. Three independent mitochondrial isolations were performed starting from an *A. thaliana* liquid cell culture. Proteins of a mitochondrial fraction were extracted, purified using the SP3 workflow, and subsequently digested separately with six different proteases. Generated peptides were analyzed by LC–IMS–MS/MS on a timsTOF Pro mass spectrometer. A total of 72 LC–MS measurements were performed (3 samples * 6 digests * 4 TIMS fractions). All raw data were finally combined and analyzed in MaxQuant (MQ) to achieve deep coverage of the mitochondrial proteome. The resulting dataset was used to comprehensively characterize the mitochondrial proteins of Arabidopsis. *Abbreviations:* SP3, single-pot, solid-phase-enhanced sample preparation; LC–IMS–MS/MS, liquid chromatography–ion mobility spectrometry–tandem mass spectrometry; Chymo, chymotrypsin; TIMS, trapped ion mobility spectrometry; TOF, time-of-flight. Red: Arg-C; Yellow: Trypsin; Blue: Lys-C; Green: Asp-N; Purple: Chymotrypsin; Gray: Glu-C.

To analyze the mitochondrial proteome of the model plant *A. thaliana* at a particular depth, we followed the experimental strategy summarized in Fig. 1. We used a nongreen *A. thaliana* cell culture as the starting material to obtain mitochondria in sufficient quantity and purity. This material corresponds exactly to the one used for a characterization of the proteome of a single Arabidopsis mitochondrion (Fuchs et al. 2020). Three independent mitochondria isolations were performed. Proteins of all three isolations were extracted, purified, and processed via the single-pot, solid-phase-enhanced sample preparation (SP3) protocol (Hughes et al. 2019; Mikulášek et al. 2021). At the center of the processing is the parallel digestion of mitochondrial fractions with the six different proteases Arg-C, trypsin, Lys-C, Asp-N, chymotrypsin, and Glu-C. While trypsin is the gold standard for shotgun proteomics for several reasons, the sole use of trypsin hinders capturing the proteome in its entirety since certain portions of the proteome remain inaccessible following digestion with only one single protease. To overcome these limitations, additional proteases have been tested for their suitability for mass spectrometry-based proteomics (Tsiatsiani and Heck 2015; Giansanti et al. 2016). These proteases cleave proteins at different positions. By digesting a protein fraction with multiple proteases, complementary parts of protein sequences and, thus, of a whole proteome can be covered. The use of multiple proteases in parallel has been consistently reported to enhance overall sequence coverage and increase general identification rates (Müller et al. 2010; Swaney et al. 2010; Guo et al. 2014; Giansanti et al. 2016; Kaulich et al. 2021; Sinitcyn et al. 2023).

To further optimize proteome coverage, peptides of the 3 × 6 samples were separated via trapped ion mobility spectrometry (TIMS) fractionation (Guergues et al. 2022) using four different ion mobility windows. Finally, all peptides of the resulting 72 fractions were sequenced and quantified by tandem mass spectrometry (MS/MS). The measured spectra were combined and assigned to corresponding protein sequences using an extended Arabidopsis protein database (see below) and the MaxQuant software tool (Prianichnikov et al. 2020). Finally, quantitative protein information (given as intensity-based absolute quantitation [iBAQ] values (Schwanhäusser et al. 2011)) from all samples was normalized, summarized, and averaged to allow in-depth investigation of the generated dataset.

### Database configuration

In a recent study of the proteome of a single Arabidopsis mitochondrion, an extended TAIR10 sequence database was used, which also included the sequences of organelle-encoded transcripts after RNA editing events with at least 50% efficiency; it comprised overall 35,568 protein entries (Fuchs et al. 2020). Here, we further extended this database in two respects: (i) Entries from the Araport11 database that were missing in the TAIR10 database were added. We decided to use TAIR10 combined with Araport11 instead of Araport11 alone because it has recently been shown in the course of building the Arabidopsis Peptide Atlas (Van Wijk et al. 2021) that some TAIR10 entries are missing in Araport11 but correspond to actually occurring proteins. For example, one such protein, with the accession number AT3G12012.1, is located in mitochondria according to the SUBA5 database (Hooper et al. 2014, 2017). We detected this protein across all six digests with high sequence coverage. (ii) For all mitochondrial-encoded proteins, we added permutated amino acid sequences representing all possible combinations of RNA editing sites in their edited or nonedited state (independently from RNA editing efficiency at a given site). Information on RNA editing sites was derived from Bentolila et al. (2013) and Lenz et al. (2018). Further details are provided in the Experimental Procedures section. In total, our Arabidopsis protein database contains 70,586 protein entries.

### A proteome comprising 1,339 mitochondrial proteins

Altogether, our proteome dataset comprises more than 100,000 distinct peptides with a peptide score of at least 40, as calculated by the Andromeda search engine (Cox et al. 2011) (Supplementary Table S1). A peptide was considered identified in a given sample if its peptide intensity calculated by MaxQuant was >0. The highest numbers of peptides were found in the Arg-C digests, while the Glu-C approach yielded the lowest numbers (Supplementary Fig. S1A).

The identified peptides were assembled into 4,909 distinct protein groups. In the MaxQuant software, a protein group is defined as a group of proteins that cannot be distinguished based on identified peptides (Tyanova et al. 2016). These homologous proteins can be either isoforms or different forms originating from differential splicing (corresponding to different gene models defined in Arabidopsis for multiple gene loci). A protein group was considered to be identified if the iBAQ value of the protein group was >0 (Supplementary Fig. S1B). For the following evaluations, we only accepted proteins that have been identified in all three biological replicates of at least one digest. This was the case for 4,692 protein groups, which we define as the “deep mitochondrial proteome” (Supplementary Table S2).

Mitochondrial fractions may include proteins from other subcellular compartments. These proteins can either be interpreted as “contaminants” or candidates that bind to mitochondria for biological reasons (Møller et al. 2021). The subcellular location database for Arabidopsis proteins (SUBA5) was used to evaluate the “deep mitochondrial proteome”. The SUBA5 portal (https://suba.live/) provides results of the SUBAcon algorithm, which integrates experimental and predictive evidence for subcellular localization of all Arabidopsis proteins (Hooper et al. 2014, 2017). In case a protein group consists of more than one protein, the protein covered by the most peptides is defined by MaxQuant as the representative of this group and was used for the SUBAcon annotation. In a few cases, corresponding SUBA entries were not available because SUBA does not cover all gene models of the TAIR10 and Araport11 databases. In these cases, version numbers were manually changed into .1 (e.g. AT3G20000.2 [TOM40] was replaced by AT3G20000.1 to perform SUBA evaluation).

**Figure 2.**
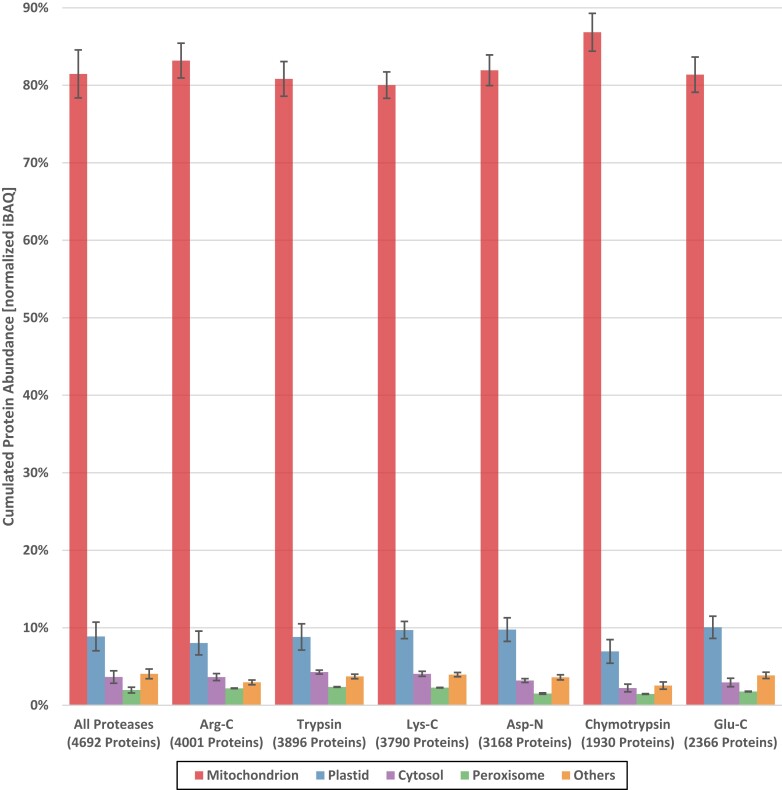
Purity estimation of the mitochondrial fraction. All proteins identified by our six-protease-proteome-approach were assigned to subcellular localizations using the SUBAcon algorithm of SUBA5 (Hooper et al. 2017). Next, protein quantities (normalized iBAQ values) were summed up for each protease digest per subcellular fraction. The bar chart indicates cumulated protein abundance of the four most prominent subcellular compartments (mitochondrion, plastid, cytosol, peroxisome; all other compartments are grouped as “others”) for the six proteases. The bars on the very left indicate the composition of the analyzed mitochondrial fraction based on combing the information from all six protease digests. Values in parentheses indicate the number of proteins identified per protease digest (number of proteins with iBAQ values >0 in all three replicates). Error bars indicate the standard deviation of the calculated protein abundance between the three replicates.

Out of 4,692 protein groups, 1,339 are annotated to be located in mitochondria, according to SUBAcon, representing 28.5% of all identified protein groups. Most of the remaining proteins were annotated to be located in plastids (989; 21%) or in the cytosol (754; 16.0%). One hundred and seventy-five protein groups (3.7%) were assigned to more than one subcellular localization. Of these, 31 protein groups were also assigned to mitochondria. Merely looking at the number of proteins identified, however, does not take into account the quantity of proteins and their quantitative contribution to the proteome. A quantitative evaluation of the proteome composition of the six different digests using normalized iBAQ values and SUBAcon revealed a purity of our mitochondrial fraction in the range of 81% (Fig. 2), which is similar to the results achieved previously (Fuchs et al. 2020).

### Proteome dynamics

**Figure 3.**
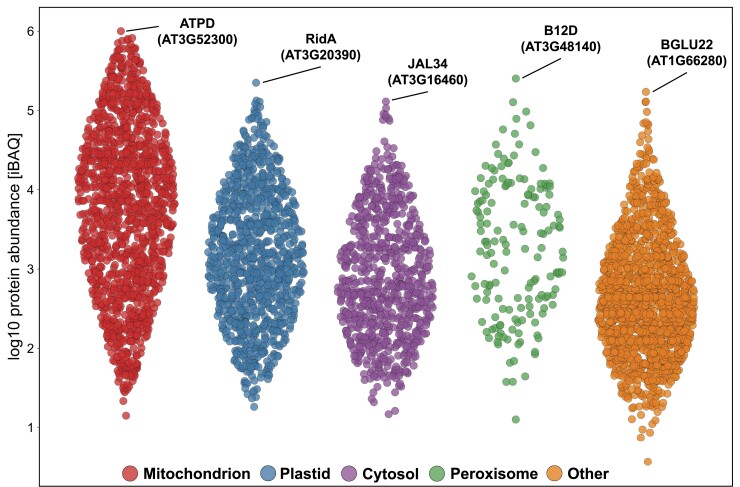
Dynamic range of proteome expression. Proteins are grouped by subcellular assignment as introduced in Fig. 2 and sorted by their log_10_-transformed abundance (based on iBAQ). Name and AGI of the most abundant protein of each assignment is given. Note that highest abundance (normalized iBAQ value) of a mitochondrial protein (the d subunit of the mitochondrial ATP synthase) is 1,000,000 (10^6^); while the highest abundance of a putative nonmitochondrial protein (the peroxisomal B12D protein) is 250,000 (10^5.4^). For B12D, both MULocDeep (Jiang et al. 2023) and DeepMito (Savojardo et al. 2020) predict localization in the mitochondrial inner membrane or matrix. Therefore, B12D is likely to be a (dual-targeted) mitochondrial protein. Figure generated with Instant Clue (Nolte et al. 2018).

The dynamic range of the 4,692 identified proteins spanned six orders of magnitude (Fig. 3). Most of the highly abundant proteins are localized in the mitochondria, according to SUBAcon, with the two most abundant nonmitochondrial proteins being assigned to peroxisomes and plastids. We also detected several mitochondrial proteins at the lower end of the abundance distribution, e.g. components of the RNA editing machinery, which are often present in low copy numbers, as reported previously (Fuchs et al. 2020). We conclude that our dataset comprehensively covers mitochondrial proteins of very different abundance classes, allowing a deep exploration of our mitochondrial fraction.

### The “nontryptic subproteome” comprises 462 proteins

**Figure 4.**
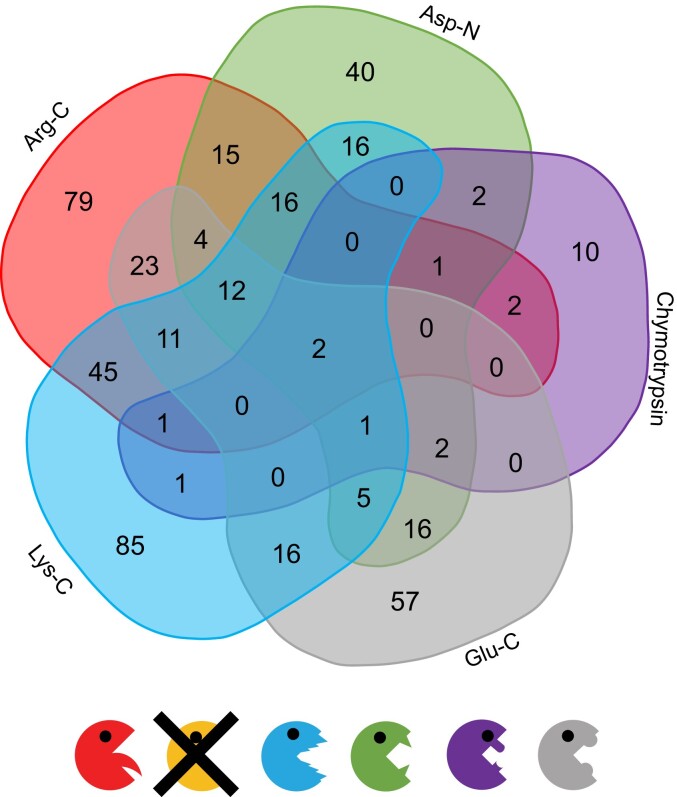
Composition of the nontryptic Arabidopsis mitochondrial subproteome. In total, 462 proteins were detected, for which no iBAQ was calculated in any of the three tryptic samples. The Venn diagram shows how many of these nontryptic proteins have been identified exclusively in each digest, as well as how many proteins have been identified in multiple digests. Most exclusive proteins have been identified in the Arg-C and Lyc-C approaches, the fewest in the Chymotrypsin approach. For each digest, we only considered protein groups with an iBAQ > 0 in 3/3 replicates. The Venn diagram was generated online at https://bioinformatics.psb.ugent.be/webtools/Venn/. Red: Arg-C; Yellow: Trypsin; Blue: Lys-C; Green: Asp-N; Purple: Chymotrypsin; Grey: Glu-C.

Next, we evaluated all protein groups that did not have an iBAQ >0 in any of the trypsin samples. This potential “nontryptic subproteome” consists of 462 protein groups. Most of these protein groups were identified in the Lys-C and Arg-C digests. These proteases cleave proteins at positions similar to trypsin (Fig. 4). We conclude that the parallel use of multiple proteases improved the coverage of the Arabidopsis mitochondrial proteome, as previously reported for the yeast and human proteome (Swaney et al. 2010; Guo et al. 2014; Sinitcyn et al. 2023). Of the 462 nontryptic protein groups, 40 (8.6%) were assigned to be located within mitochondria, while most of the predicted nonmitochondrial protein groups were assigned to the nucleus (134; 29%), the cytosol (93; 20%), and plastids (65; 14%). A quantitative evaluation of the “nontryptic subproteome” revealed that most of the protein groups are of lower abundance within the whole dataset, matching observations made in yeast (Swaney et al. 2010). Among the most abundant proteins not detected in our trypsin samples were the succinate dehydrogenase subunit 8 (SDH8; AT2G46390.1) and a Cox19 family protein (AT1G02160.1), both rather short proteins of only 46 and 71 amino acids, respectively, that can be identified by peptides generated by chymotrypsin and Asp-N digestion. This result is consistent with previous observations that the use of multiple proteases can especially improve the identification of small proteins or short open reading frame-encoded peptides (SEPs) (Kaulich et al. 2021).

### Multiprotease digestion boosts protein coverage

Proteome analysis using multiple proteases not only improves the identification of particularly small and low-abundant proteins but also offers a greatly increased coverage of proteins by identified peptides (Tsiatsiani and Heck 2015). This increase in coverage can promote the identification of PTMs and allow the differentiation of protein species with high sequence similarity, for example, proteins generated by translating differentially spliced or edited transcripts.

To evaluate the increase in protein sequence coverage from the combined use of six different proteases, we compared the results of our current study with those of a recently published previous study (Fuchs et al. 2020) (Supplementary Fig. S2). A direct comparison of our tryptic dataset with that of Fuchs et al. (2020) indicates that upgrading to a faster and more sensitive mass-spectrometer and using the more efficient SP3-based sample preparation method instead of classical in-gel digestion leads to the identification of more than 1,300 additional protein groups. However, the overall sequence coverage of the identified protein groups is comparable (Supplementary Fig. S2, left part and center). Careful inspection of the data even reveals a slightly reduced sequence coverage in our current study. We assume that the increased efficiency of our tryptic digestion leads to a reduction of missed cleavage sites, resulting in smaller peptide species, causing a slightly reduced coverage of the respective parent proteins. In the two compared studies, up to two missed cleavage sites per protein were allowed for trypsin-digested samples, and a false discovery rate (FDR) of 1% was applied at the peptide spectrum match (PSM) and protein level. In the approach presented here, 83% of the peptides were free of missed cleavages, compared to 68% in the study reported by Fuchs et al. (2020).

**Figure 5.**
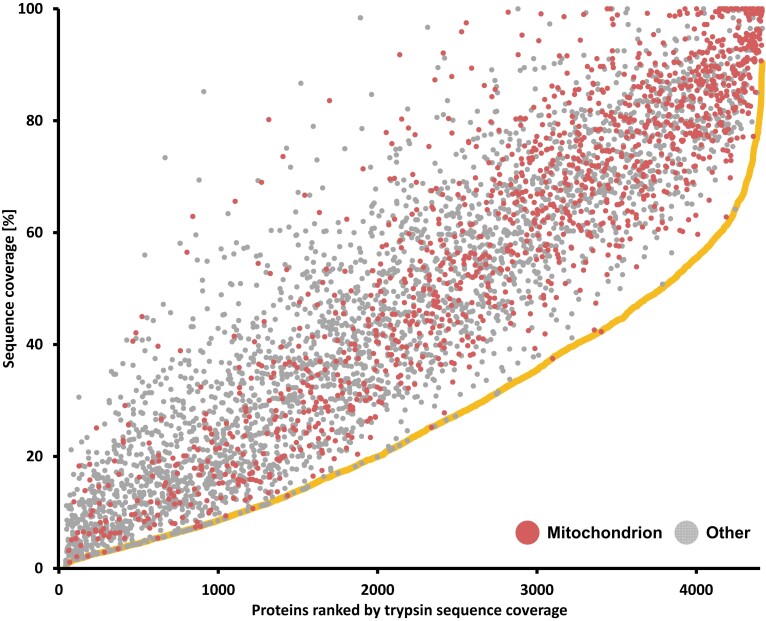
Comparison of sequence coverage for each of the identified proteins by tryptic peptides and by combined peptides of all proteases. Proteins are ranked by their sequence coverage by tryptic peptides (yellow line). Dots show the sequence coverage by the combined protease digests including trypsin. Red dots show proteins assigned to Mitochondria by SUBA, gray dots show proteins assigned to other compartments. The greater the distance of a dot from the yellow line, the greater the gain in sequence coverage.

However, the sequence coverage substantially increases upon proteome analysis using additional proteases (Supplementary Fig. S2, right part). A comparison of our tryptic with our multiprotease dataset showed that increased sequence coverage was achieved for all but 99 mostly low-abundant proteins. The increase varied depending on the protein (Fig. 5). Nearly half of all protein groups had a sequence coverage of ≥50%, and 30 protein groups were covered by 100%. The average sequence coverage of the 1,339 proteins assigned to mitochondria by SUBA5 was 60.9%. This sequence coverage provides the ability to systematically follow the consequences of RNA editing and differential splicing at the protein level in great depth.

### Consequences of RNA editing on the protein level: detection of peptides corresponding to nonedited transcripts

A recently reported ribosome profiling analysis in Arabidopsis revealed that partially edited mitochondrial transcripts are not entirely degraded before translation and that their respective editing levels showed an increase when loaded on mitoribosomes (Planchard et al. 2018). The factor causing this increase in editing efficiency is yet to be identified but might be explained by better accessibility of incompletely edited transcripts to the editing machinery since several RNA editing factors were found in fractions containing mitoribosomes (Rugen et al. 2019). Another explanation for this observation might be that mitoribosomes preferably bind transcripts that already have a high editing status (Small et al. 2020). While partially edited transcripts are translated to a non-negligible extent, previous studies indicate that OXPHOS subunits transcribed from nonedited transcripts do not accumulate due to yet unknown post- or co-translational processes (Grohmann et al. 1994; Lu and Hanson 1994). Interestingly, a protein polymorphism generated by the translation of differentially edited transcripts was shown for rRPS12 from petunia and maize, a component of the translational machinery itself (Lu et al. 1996; Phreaner et al. 1996). It is still not known whether protein diversity generated from differential RNA editing fulfills any regulatory function (Small et al. 2020; Knoop 2022).

Most of the studies mentioned above used antibody-based methods to detect edited or unedited protein species since modern mass spectrometry-based approaches were not yet available. We, therefore, re-investigated this issue in Arabidopsis using our deep mitochondrial proteome. To analyze the consequences of partial RNA editing on the proteome level, we systematically inspected our data set for protein groups containing proteins corresponding to non- or partially edited RNAs. We then manually inspected “nonedited peptides”. With “nonedited peptides,” we here refer to peptides that have been translated from a transcript that has not been edited. In consequence, these peptides still carry the amino acid encoded in the mitochondrial genome. In contrast, we refer to “edited peptides” if they were translated from edited transcripts and are thus affected by the amino acid exchange introduced by RNA editing. To ensure correct identification of non-edited peptides, they had to fulfill certain criteria, which are described in the “Experimental Procedures” section. The results of our evaluation are summarized in Supplementary Table S3; all annotated spectra can be inspected in MS-Viewer (Baker and Chalkley 2014; see the data availability statement at the end of our article). The mitochondrially encoded proteins identified in the course of our investigation carry 302 amino acid positions, which could be affected by RNA editing. 103 of these sites were covered by peptides. Overall, only a few nonedited peptides could be detected. The corresponding proteins are described below.

Consistent with previous results, we did not detect nonedited peptides of respiratory chain subunits, with one exception. This is expected because the editing extent of the corresponding transcripts is very high, most likely reflecting that the amino acid exchanges caused by RNA editing are crucial for the assembly, structure and correct function of the respiratory chain complexes (Bentolila et al. 2013; Maldonado et al. 2022). The only exception is a low abundant nonedited peptide covering the editing site “atp4eU416TIp98” (Supplementary Fig. S3) in the ATP4 subunit (ATMG00640) of mitochondrial complex V [“atp4eU416TIp98”: This nomenclature of RNA editing sites was proposed by Rüdinger et al. (2009) and reads as follows: The nucleotide “U” results from editing of the original “C” at the position 416 of the corresponding mRNA. Counting starts with the first nucleotide of the AUG start codon. Upon translation, the edited peptide carries isoleucine (I), while the nonedited peptide carries a threonine (T). Editing efficiency at this position has been reported to be 98% (Bentolila et al. 2013)].

Furthermore, nonedited peptides were detected for several mitoribosomal proteins. We investigated the editing status of the RPS12 protein (ATMG00980) to test whether we could observe the protein polymorphism described in petunia and maize in *A. thaliana*. With our approach, we were able to cover five out of seven RNA editing sites. Despite an editing extent of 0.79 to 0.91, all sites were exclusively detected in their edited state. We conclude that the nonedited protein is not detectable, which is in contrast to the observations reported for suspension cell culture mitochondria from petunia and maize shoots (Lu et al. 1996; Phreaner et al. 1996).

We furthermore investigated the editing status of RPL5 (ATMG00210). All editing sites of this protein are covered by peptides of our deep mitochondrial proteome dataset. The editing rate of RPL5 (ATMG00210) transcripts covers a high range from 0.03 for rpl5eU121PSp3 to 0.95 for rpl5eU329SLp95. Especially for rpl5eU121PSp3, a high level of nonedited peptides is expected since only 3% of the transcripts are edited. Interestingly, in addition to the nonedited peptides, we were also able to detect the edited peptides, but with lower peptide intensity (Supplementary Fig. S4), indicating that edited proteins do accumulate despite very low transcript levels. For all other RNA editing sites, we only detected edited peptides.

RPS3 (ATMG00090) also has three editing sites with low editing frequency: rps3eU187LFp2, rps3eU515SLp6, and rps3eU1580SFp11. Peptides covering rps3eU187LFp2 or rps3eU515SLp6 were not detected, neither the edited nor the nonedited version. Site rps3eU1580SFp11 is surrounded by sites rps3eU1571AVp97 and rps3eU1598SLp97, with a high editing efficiency of 97%. Our permutation database (see Experimental Procedures) allowed us to further explore this region of RPS3, as it contains all possible peptide versions. The best-scoring peptide covering all three sites was detected in the Lys-C digests and indicates that the most abundant variant of the RPS3 protein carries the amino acid exchanges introduced at sites rps3eU1571AVp97 and rps3eU1598SLp97 but not at site rps3eU1580SFp11 (Supplementary Fig. S5). We further detected peptides indicative of a protein polymorphism at amino acid 296 corresponding to RNA editing site rps3eU887SLp73 (Supplementary Fig. S6).

### Nonedited proteins are present in subunits of native protein complexes

Are proteins derived from non- or partially edited mitochondrial transcripts assembled into mitochondrial protein complexes and functional? To answer this question, we used our results to re-investigate complexome profiling datasets of mitochondria of Arabidopsis leaves (overall 10 datasets; Rugen et al. 2021, PRIDE Identifier: PXD019253). Complexome profiling is based on the native separation of protein complexes, most often using Blue native PAGE (Arnold and Braun 2022). A lane of the native gel is subsequently cut into small slices from bottom to top. All slices are finally subjected to label-free quantitative shotgun proteomics. This experimental approach allows the systematical profiling of protein complexes of a biochemical fraction of interest. Re-evaluation of the complexome profiling datasets for Arabidopsis leaves using our extended protein database indeed allowed the detection of nonedited peptides (Supplementary Table S3).

**Figure 6.**
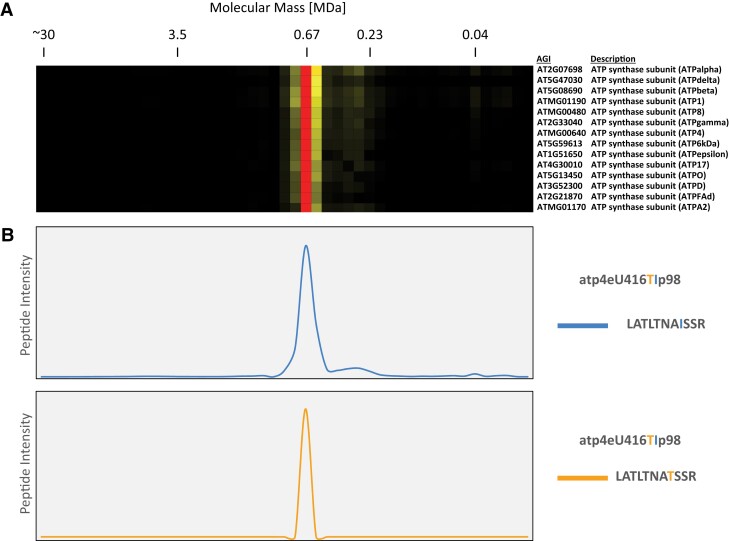
Abundance profiles of editing-specific peptides in the mitochondrial ATP synthase complex upon large-pore complexome profiling analysis. Mitochondrial protein complexes were resolved on a one-dimensional large pore Blue-native gel as described previously (Rugen et al. 2021). The resulting gel lane was dissected into 46 gel pieces from top to bottom and used for label-free quantitative shotgun proteome analyses as described before (Rugen et al. 2021). **A)** Heatmap of clustered proteins belonging to the mitochondrial ATP synthase complex (original data from Rugen et al. 2021). Each row of the heatmap indicates abundance of a single protein (accession number and name to the right) along the Blue-native gel lane (rows were hierarchically clustered). Color indicates protein abundance and is normalized for each protein individually: Red: maximum, light yellow: minimum; black: no detection. A molecular mass standard is given above the heatmap. **B)** Abundance profiles of two peptides of the ATP4 subunit (encoded by ATMG00640) along the lane of the large pore Blue-native gel. Both peptides are generated by translation of regions of the ATP4 mRNA that carry the RNA editing site atp4eU416TIp98. The editing site is labeled according to Rüdinger et al. (2009): atp4eU416TIp98—The nucleotide “U” results from editing of the original “C” at the position 416 of the corresponding mRNA; counting starts with the first nucleotide of the AUG start codon; upon translation, the edited protein carries Isoleucine (I) instead of Threonine (T); editing efficiency has been reported to be 98%. Blue graph: Abundance profile of the edited peptide. Orange graph: Abundance profile of the nonedited peptide. Editing efficiency as reported by Bentolila et al. (2013). *y*-Axis not at the same scale.

Consistent with our deep mitochondrial proteome results, our complexome datasets include two peptides of the ATP4 subunit (ATMG00640) covering the editing site “atp4eU416TIp98” (Supplementary Fig. S7). The more abundant peptide corresponds to the edited ATP4 transcript, the other to the nonedited transcript. Interestingly, both peptides peak at 600 kDa in our complexome profile and are included in the cluster of the subunits of the ATP synthase complex (Fig. 6). We conclude that ATP4 subunits edited and not edited at editing site atp4eU416TIp98 are assembled into the native ATP synthase complex. Based on a comparison of peptide intensity, less than 1% of all ATP synthase complexes carry the nonedited protein. This corresponds to the high percentage of edited transcripts of 98% (Bentolila et al. 2013).

**Figure 7.**
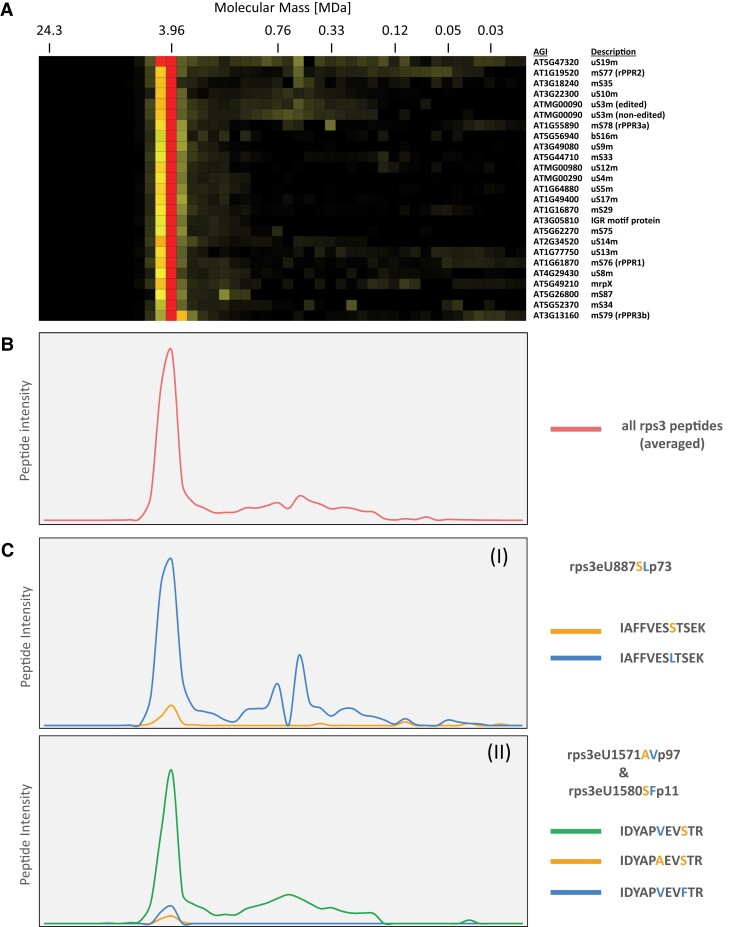
Abundance profiles of editing-specific peptides in the small mitoribosomal subunit upon large-pore complexome profiling analysis. Experimental approach: see the legend of Fig. 6. **A)** Heatmap of clustered proteins belonging to the small mitoribosomal subunit (original data from Rugen et al. (2021), classification of mitoribosomal proteins according to Rugen et al. (2019) and Waltz et al. (2019)). Each row of the heatmap indicates abundance of a single protein (accession number and name to the right) along the Blue-native gel lane (rows were hierarchically clustered). Color indicates protein abundance and is normalized for each protein individually: Red: maximum, light yellow: minimum; black: no detection). A molecular mass standard is given above the heatmap. **B)** Averaged abundance profile of all RPS3 peptides along the lane of the large pore Blue native gel. **C)** Abundance profiles of editing-specific peptides of the RPS3 subunit (encoded by AtMg00090) along the lane of the large pore Blue native gel. Editing sites are labeled according to Rüdinger et al. (2009): (I) rps3eU887SLp73—The nucleotide “U” results from editing of the original “C” at the position 887 of the corresponding mRNA; counting starts with the first nucleotide of the AUG start codon; upon translation, the edited peptide carries Leucine (L), while the nonedited peptide carries a Serine (S); editing efficiency has been reported to be 73% (Bentolila et al. 2013). Orange graph: abundance profile of the nonedited peptide; blue graph: abundance profile of the edited peptide; (II) rps3eU1571AVp97/rps3eU1580SFp11—The mRNA is edited at two sites in the region encoding this peptide, resulting in possible Alanine (A) to Valine (V) and/or Serine (S) to Phenylalanine (F) exchanges (editing efficiencies are 97% and 11%). Orange graph: Abundance profile of the fully nonedited peptide; blue graph: Abundance profile of the fully edited peptide; Green: Abundance profile of a peptide edited at rps3eU1571AVp97 but not at rps3eU1580SFp11. Editing efficiency as reported by Bentolila et al. (2013).

Also, for RPS3 (ATMG00090), peptides derived from nonedited transcripts were detected in our complexome profiling datasets (Fig. 7, Supplementary Figs. S8 and S9). Peptides covering editing site rps3eU887SLp73 were present in both edited and nonedited forms. In the mitochondrial complexome profiles, they peak at 4 MDa and cluster together with the proteins of the small mitoribosomal subunit (mtSSU, Fig. 7C (I)). We conclude that RPS3 is assembled into the native ribosome regardless of the editing state at rps3eU887SLp73. According to peptide intensity values, 9% of mtSSU complexes carry the RPS3 protein not edited at site rps3eU887SLp73, whereas 27% of the corresponding transcripts are not edited (Bentolila et al. 2013), and 12% of the transcripts after loading on ribosomes (Planchard et al. 2018). This indicates an increase in the editing event at this site during translation.

In addition, we evaluated peptides of RPS3 covering the closely spaced editing sites rps3eU1571AVp97, rps3eU1580SFp11, and rps3eU1598SLp97 in our complexome profiling datasets. Two of the three sites, rps3eU1571AVp97 and rps3eU1580SFp11, are located on a tryptic peptide we detected. Three versions of this peptide are present: (i) both sites edited, (ii) both sites not edited, and (iii) the combination of edited rps3eU1571AVp97 and nonedited rps3eU1580SFp11, nicely reflecting the editing efficiency at the two sites (Supplementary Fig. S10). All three peptide versions are present in the complexome fractions containing the mtSSU (Fig. 7C (II)). The peptide derived from an RPS3 transcript edited at rps3eU1571AVp97 but not edited at rps3eU1580SFp11 is the most abundant within the native ribosomal subunit (86% according to peptide intensity), while the peptide edited at both sites accounted for 10% and the peptide not edited at both sites for 4%. For the two editing sites, this results in 96% editing at rps3eU1571AVp97 and 10% at rps3eU1580SFp11, which is in the range of the editing levels reported for the corresponding transcripts (Bentolila et al. 2013).

### Characterization of alternative splicing events on the proteome level

Several transcripts in Arabidopsis undergo alternative splicing (AS). AS may greatly increase the proteome complexity. Forms of a protein generated by differential splicing may have varying molecular functions, interact with different proteins, nucleic acids or membranes and may have different subcellular localizations (Kelemen et al. 2013). AS can also cause the downregulation of a gene by disruption of the open reading frame (ORF) of its transcript, thereby causing biosynthesis of a truncated protein or initiating a nonsense-mediated decay (NMD) response (Lewis et al. 2003). In Arabidopsis, it is estimated that >60% of all multiexonic transcripts are alternatively spliced through various AS events across developmental stages and in different tissues (Martín et al. 2021).

In the course of our study, we identified 46 protein groups derived from 23 genes, which may originate from AS (Supplementary Table S4). Among the most abundant protein groups are the 40 kDa subunit of the preprotein translocase of the outer membrane (TOM40-1) (AT3G20000.1), prohibitin 2 (AT1G03860.3/AT1G03860.1), the E2 Subunit of the oxoglutarate dehydrogenase complex (OGDC-E2) (AT4G26910.2/AT4G26910.1), and the γCA3 subunit of complex I (AT5G66510.1). For further evaluation, we focused on proteins that were especially well covered by identified peptides. Seventeen mitochondrial protein groups had a coverage of >70% in our dataset. We manually examined unique peptides of the splice variants. The inclusion of peptides in our evaluation required high-quality MS/MS spectra in at least two samples. In the following sections, we discuss the results for TOM40-1 and OGDC-E2.

**Figure 8.**
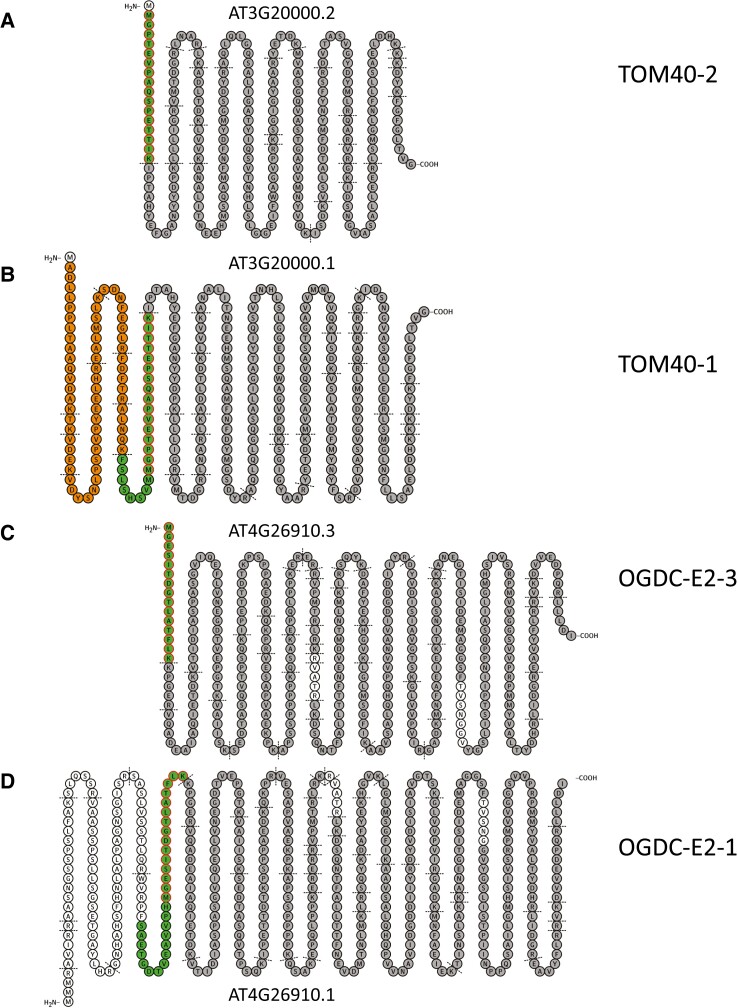
Sequence comparison of two proteoforms of the 40 kDa subunit of the preprotein translocase of the outer mitochondrial membrane (TOM) the E2 subunit of the oxoglutarate dehydrogenase complex (OGDC), respectively. Amino acid sequences are arranged using the Protter software tool (Omasits et al. 2014). Horizontal black lines indicate cleavage sites for trypsin. **A, B)** Proteoforms of TOM40. Two tryptic peptides unique to one or the other proteoform are given in green (the shorter peptide in TOM40-1 is the result of removed exons during splicing). Peptides only present in TOM40-1 are given in orange; peptides present in both proteoforms in gray; nondetected peptides in light gray. **C, D)** Proteforms of OGDC-E2. Two tryptic peptides unique to one or the other proteoform are given in green (the shorter peptide in OGDCE2-3 is the result of removed exons during splicing). Peptides present in both proteoforms in gray; nondetected peptides in light gray.

For TOM40-1, splicing of the AT3G20000 pre-mRNA leads to two different transcripts, AT3G20000.1 and AT3G20000.2, which differ at the N-terminus. The identified N-terminal semitryptic peptide of the shorter protein variant (encoded by AT3G20000.2) comprises 16 amino acids (Fig. 8, Supplementary Fig. S11). The corresponding tryptic peptide of the longer protein variant (encoded by AT3G20000.1) starts with the same amino acid sequence but is extended by eight amino acids. Furthermore, the N-terminus of this protein variant is extended by another 64 amino acids, all of which are covered by the detected peptides (Fig. 8; Supplementary Fig. S11). The location of both proteins is predicted to be the outer mitochondrial membrane, according to MULocDeep (Jiang et al. 2023).

For OGDC-E2, we detected peptides for transcripts AT4G26910.1 and AT4G26910.3. Again, the two corresponding protein variants differ in their N-terminus (Fig. 8). The identified semitryptic peptide of the N-terminus of the shorter protein variant includes 15 amino acids. The corresponding sequence region of the longer protein variant is covered in our chymotrypsin and AspN digests (Supplementary Fig. S12). The peptides of these digests include the sequence of the tryptic peptide of the shorter protein variant but are extended. The larger protein variant encoded by AT4G26910.3 is even further extended at the N-terminus, but no corresponding peptides were detected. While MULocDeep predicts localization in the mitochondrial matrix for AT4G26910.1, it predicts localization in the cytosol for AT4G26910.3. Analysis of the N-termini for possible signal peptides or mitochondrial transfer peptides using TargetP (Armenteros et al. 2019) revealed that the shorter AT4G26910.3 does not carry a mitochondrial transfer peptide that is predicted for the longer AT4G26910.1.

### Proteome explorer: a tool to explore the “deep mitochondrial proteome”

Our protein identification and quantification data are provided in Supplementary tables accompanying this publication (Supplementary Tables S1 and S2). To facilitate the use and accessibility of our primary data, we have developed an online data visualization tool, the “Proteome Explorer”, which directly links extensive protein and peptide information to a “Proteomap”.

**Figure 9.**
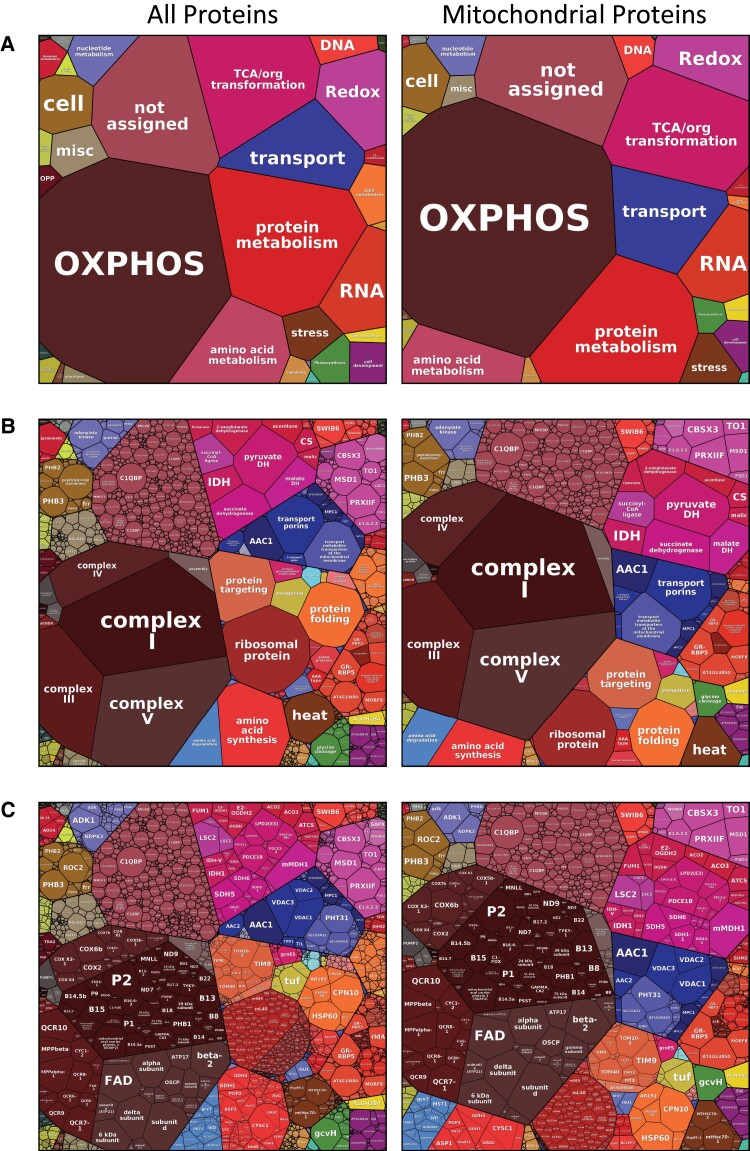
Proteomaps of the Arabidopsis mitochondrial fraction. Proteins are grouped according to functional categories (**A**) or more detailed functional subcategories, e.g. affiliation with a protein complex (**B** + **C**). The proteomaps were generated at the proteomaps portal (https://www.proteomaps.net/; Liebermeister et al. 2014). In all three proteomaps, each protein is represented by a polygon whose size reflects the abundance according to its iBAQ value. Assignment of proteins to functional categories according to Heinemann et al. (2021). Functional information on a few known mitochondrial proteins not assigned to categories in this reference was manually supplemented. Left column: proteomap showing all proteins identified; right column: proteomap only showing proteins assigned to mitochondria by the SUBA5 database (https://suba.live/index.html). Both data sets can be fully explored in Proteome Explorer (www.proteomeexplorer.de).

“Proteomaps” provide an elegant visualization method for a clear overview of complex datasets, allowing conclusions about their functional composition (Liebermeister et al. 2014). In a proteomap, each protein is represented by a polygon whose size corresponds to the relative proportion of the protein in the total proteome. Functionally related proteins are situated in adjacent regions and can be further labeled with similar colors. Proteomaps of the full multiprotease dataset, as well as a dataset only containing mitochondrial and dual-targeted proteins (according to SUBA5), are presented in Fig. 9, illustrating that proteins belonging to the OXPHOS system represent the most abundant category, accounting for nearly 30% of all protein abundance in our mitochondrial fraction, while TCA cycle-related proteins represent ∼9%, and the mitoribosome ∼3.3%. Since the quantification of splice variants and RNA editing proteoforms by iBAQ is not very precise, we summed the iBAQs of all “ProteinGroups” of each gene and only showed the sequence of the best-scoring proteoform. Spectra of all identified proteins can still be examined via MS-Viewer (Baker and Chalkley 2014).

In order to make our extensive proteome datasets as accessible as possible, we looked for a way to link the large amount of information in our Supplementary tables to the more concise proteomaps. Since the quantitative proteomaps from Liebermeister et al. (2014) do not support the attachment and presentation of supplementary data, a tool was developed to integrate and publish proteomaps with accompanying protein and peptide information. The “Proteome Explorer” web platform allows users to upload three files: a peptide table containing information such as peptide sequence, starting position in the protein sequence, and protease data used for peptide identification; a protein table containing information such as annotation, coverage, protein sequence, and localization (determined by SUBAcon); and the largest SVG file exported from proteomaps, containing all positional data for the map.

To reduce bandwidth requirements and improve loading times for Proteome Explorer, a PHP script is used to extract node information from the particularly large SVG file and make it available to the application as JSON. Protein and peptide information are provided as tab-separated values, which can be easily read and processed by JavaScript. The website initially displays the proteomap in its exported form, but as the user hovers over a node with the mouse, additional information about the selected protein is displayed, while other proteins are slightly dimmed. Additionally, the protein's cluster and higher-order clusters are highlighted. By clicking on the protein, the information window becomes permanent, allowing users to access the sequence explorer or examine other nodes.

A search function is available next to the map, allowing users to search for AGI, protein name, or localization. Search terms separated by spaces are combined using AND logic. A category term can be prepended to a search term to filter specific data. Search assistance and examples are provided on the page. When results are found, they are automatically highlighted on the map, allowing users to display, for example, all mitochondrial proteins using “subacon:mitochondrion”.

The Sequence Explorer window allows users to inspect sequence information for individual proteins and their peptides. The main protein of each protein group is searched across all protein IDs in the peptide table, and matching peptides are aligned to the amino acid sequence of the main protein. Since certain peptides were only found during digestion with specific proteases in the experiment, controls were incorporated to filter based on this feature. Peptides are arranged as compactly as possible to enhance clarity. Users can hover over peptides with the mouse to access additional information such as mass, charges, or the number of missed cleavages for each protease. In addition, we have deposited all our annotated MS/MS spectra on MS-Viewer (Baker and Chalkley 2014). To simplify the search and inspection for peptide spectra of interest, all spectra are directly accessible via Proteome Explorer by clicking on one of the corresponding Andromeda Peptide Scores in the Sequence Explorer. The user is then automatically redirected to the corresponding spectrum in MS-Viewer. Proteome Explorer does not rely on outputs from MaxQuant but can also process data from other popular programs such as MSFragger, Proteome Discoverer or PEAKS.

Proteome Explorer, along with a video demonstrating its various functions, is available at https://proteomeexplorer.de.

The multiprotease dataset presented here can be explored via Proteome Explorer using the following link: https://proteomeexplorer.de/2.

We also provide a map that considers only proteins assigned to mitochondria by SUBA5: https://proteomeexplorer.de/3/.

## Conclusions & outlook

By applying a deep shotgun proteomics strategy, we present the mitochondrial proteome of *A. thaliana* at considerable coverage. For numerous proteins, we show that their identification and quantification are made possible by the use of proteases other than trypsin. Of the 4,692 identified proteins, 1,339 proteins are assigned to the mitochondria according to the SUBAcon algorithm. It can be assumed that more of the identified proteins are present in the mitochondria. The mitochondrial proteins vary widely in their abundance, e.g. there are highly abundant OXPHOS proteins and very low abundant RNA editing factors. We conclude that our dataset comprehensively covers the mitochondrial proteome.

The high sequence coverage of our approach allowed us to characterize more than 100 RNA editing sites at the protein level. For most of these sites, we found amino acids matching the edited transcripts, implying that proteins are predominantly translated from completely edited transcripts independent of the editing rates at the individual sites. Since complete editing of a transcript is not a prerequisite for ribosome loading (Planchard et al. 2018), we assume co- or post-translational processes that prevent the accumulation of partially edited proteins. At the same time, some peptides matching nonedited transcripts also have been found. By re-analyzing a mitochondrial complexome profiling dataset, we show that partially edited RPS3 and ATP4 proteins are incorporated into the small mitoribosomal subunit and the mitochondrial ATP synthase complex, implicating the existence of heterogeneous mitoribosome and ATP synthase populations.

**Figure 10.**
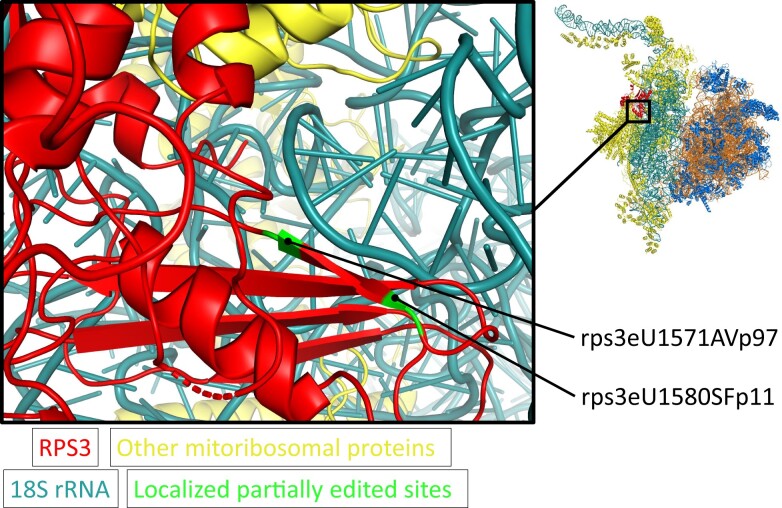
Localization of peptides of the RPS3 protein translated from nonedited transcripts (Fig. 7) in the structure of the small mitoribosomal subunit from plants. Structural data from Electron Microscopy Data Bank (EMDB-10654) (Waltz et al. 2020). The entire plant mitoribosome is shown to the right. The region enlarged to the left is indicated. Cyan: 18S rRNA; Red: RPS3; Green: positions in the RPS3 protein in which partial RNA editing leads to amino acid exchanges. The structure was analyzed with PyMOL (The PyMOL Molecular Graphics System, Version 2.5.4 Schrödinger, LLC). A PyMOL showfile allowing exploration of the structure in 3D is attached to this manuscript (Supplementary Data Set 1).

To evaluate this aspect, we visualized the RPS3 peptides corresponding to the nonedited transcripts within the recently published atomic model of the plant mitoribosome (Waltz et al. 2020). Since this model does not completely cover RPS3, only editing sites rps3eU1571AVp97 and rps3eU1580SFp11 could be located in the structure (Fig. 10, Supplementary Data Set 1). It becomes apparent that the amino acid positions corresponding to the two editing sites are not located at the surface but inside the small mitoribosomal subunit. We conclude that the different RPS3 variants cannot be involved in regulating mitoribosomal activity by the binding of external factors. The two amino acid positions are located in a four-strand beta sheet that is in close proximity to the 18S rRNA. Variation of amino acids at this site due to partial RNA editing could affect the interaction of RPS3 with the 18S rRNA, which should be further investigated. The 250 amino acid stretch of RPS3, which is missing in the mitoribosome structure and which includes the editing site rps3eU887SLp73, is most likely part of a large density at the “head” of the small subunit (Waltz et al. 2020). This indicates that the amino acid position corresponding to rps3eU887SLp73 is likely to be located on the outside of the mitoribosome.

The ATP4 subunit (also known as subunit b of ATP synthase or ATPb in Arabidopsis or Atp4p in yeast) is located in the peripheral stalk domain of the ATP synthase complex (Röhricht et al. 2021). Since there is no high-resolution structure available for the mitochondrial ATP synthase from *A. thaliana*, we mapped the position of RNA editing site atp4eU416TIp98 in the structure of yeast ATP synthase (Protein Data Bank entry 6CP6, Srivastava et al. 2018) to approximately amino acid position 190 in the homologous yeast Atp4p subunit based on a sequence alignment. This position is in close proximity to yeast Atp5p (which corresponds to Arabidopsis OSCP, At5g13450) and yeast ATP synthase subunit H, which is located at a position of the ATP-Fad subunit (At2g21870) in Arabidopsis (Röhricht et al. 2021). Based on the yeast structure, we would, therefore, localize atp4eU416TIp98 to the outside of ATP synthase and assume that it would be accessible to external regulators from the mitochondrial matrix.

The biological implications of protein heterogeneity caused by differential editing remain to be established. The rare ATP4 form containing a peptide derived from a nonedited transcript site may reflect insufficient quality control during translation. However, for other sites, this does not seem to be the case. The proportion of RPS3 derived from partially edited transcripts is higher, e.g. 9% for site rps3eU887SLp73. We currently cannot rule out that proteins derived from non- or partially edited transcripts are dysfunctional, but consider this to be unlikely, at least for the ribosomal proteins, since their proportion within native ribosomes is relatively remarkable. A possible regulatory role of differential editing in the mitochondria of plants should be addressed by future investigations. In the duckweed species *Spirudela polyrhiza*, variation of RNA editing of chloroplast transcripts between different developmental stages has been reported (Wang et al. 2015). Also, in maize (*Zea mays*), RNA editing efficiency has been found to vary in different developmental stages and tissues (Grosskopf and Michael Mulligan 1996). Reduced C-to-U RNA editing rates were described during the stress response in Arabidopsis and suggested to play a regulatory role (Chu and Wei 2020). The study of editing-deficient mutants could help to further elucidate the possible regulatory function of differential editing in the mitochondria of plants.

We developed the Proteome Explorer to facilitate data mining by the scientific community. In fact, we were not able to look at the 4,692 proteins covered by our study in detail. The Proteome Explorer comprehensively links proteomaps with experimental primary data and should make searching our dataset easy and attractive. The portal will be expanded to include more shotgun proteomics datasets and is open to external users.

## Materials and methods

### Plant material

A heterotrophic Arabidopsis (*A. thaliana*) Columbia 0 cell suspension culture was established, as described previously (Sunderhaus et al. 2006; Schikowsky et al. 2017). Cells were subcultured on a weekly basis, after which their mass increased by a factor of 3. Mitochondria were isolated after 7 d of culture by a combination of differential centrifugation and isopycnic Percoll (GE Healthcare, Solingen, Germany) gradient centrifugation as described before (Werhahn et al. 2001). Isolated mitochondria were washed, pelleted, and resuspended to yield a concentration of 100 mg mitochondria per mL and stored at −80 °C. Three independent mitochondria isolations were performed and used as biological replicates.

### Protein extraction, digestion, and peptide clean-up

The protein concentration of each isolate was determined via Bradford assay (Thermo Fisher Scientific) on a plate reader (Multiscan Sky, Thermo Fisher Scientific) according to the manufacturer's instructions.

Mitochondrial proteins were prepared for mass spectrometry analysis via the single-pot, solid-phase enhanced sample preparation (SP3) protocol developed by Hughes et al. (2019). Here, we used a protocol from Mikulášek et al. (2021) with minor adaptations: For each of the three mitochondria isolates, six fractions were mixed with equal volumes of 2× SDT (SDS-DTT-Tris) buffer (8% [w/v] SDS (sodium dodecyl sulfate), 0.2 M DTT (dithiothreitol), 0.2% [w/v] Tris–HCL, pH 7.6) and incubated on a thermal shaker (TS-100, Kisker Biotech, Steinfurt, Germany) for 1 h at 60 °C and 1,000 rpm. After centrifugation for 10 min at 20,000 × *g*, the supernatant was transferred into a new reaction tube and sonicated in a water bath for 10 min (Elmasonic S30, Elma, Singen, Germany) and centrifuged again for 10 min at 20,000 × *g*. From the supernatant, a volume corresponding to 100 *µ*g protein was transferred into a new reaction tube, and proteins were alkylated via incubation in 20 mM Iodoacetamide for 30 min at 600 rpm at room temperature in the dark. Alkylation was stopped by the addition of 5 mM DTT.

Sera-Mag Beads Carboxylate-Modified hydrophilic solids (GE Life Sciences) were combined 1:1 with hydrophobic solids, and a total amount of 600 *µ*g beads were added to each sample. Proteins were precipitated by the addition of 70 *µ*L ethanol (100%) and subsequent incubation for 10 min at 1,000 rpm at 24 °C. Beads were pelleted on a magnetic rack for 2  min and proteins were washed three times with 140 *µ*L of fresh 80% [v/v] ethanol. After protein clean-up, beads were transferred in fresh 80% [v/v] ethanol into low protein-binding tubes (Low Binding Micro Tubes, Sarstedt, Nümbrecht, Germany), and all ethanol was removed from the magnetic racks.

Proteins were generally digested according to the manufacturer's instructions. All proteases were purchased from Promega (Madison, WI, USA). *Trypsin:* Proteins were digested with 2 *µ*g of sequencing-grade modified Trypsin in 50 mM ammonium bicarbonate (pH 7.8) at 37 °C at 1,000 rpm overnight in a total reaction volume of 60 *µ*L. *Lys-C:* Proteins were digested with 3 *µ*g of mass spec grade rLys-C in 25 mM Tris–HCL (pH 8.5) and 1 mM EDTA at 37 °C at 1,000 rpm overnight in a total reaction volume of 60 *µ*L. *Chymotrypsin:* Proteins were digested with 3 *µ*g of sequencing-grade chymotrypsin in 100 mM Tris–HCL (pH 8.0) and 10 mM CaCl_2_ at 25 °C at 1,000 rpm overnight in a total reaction volume of 60 *µ*L. *Asp-N:* proteins were digested with 2 *µ*g of sequencing-grade Asp-N in 50 mM Tris–HCL (pH 7.5) at 37 °C at 1,000 rpm overnight in a total reaction volume of 100 *µ*L. *Glu-C:* proteins were digested with 3 *µ*g of sequencing-grade Glu-C in 45 mM ammonium bicarbonate (pH 7.5) at 37 °C at 1,000 rpm overnight in a total reaction volume of 100 *µ*L. *Arg-C:* proteins were digested with 1 *µ*g of sequencing-grade Arg-C in 50 mM Tris–HCL (pH 7.8), 2 mM EDTA, 5 mM DTT 5 mM CaCl_2_ at 37 °C at 1,000 rpm overnight in a total reaction volume of 60 *µ*L. The next day, the activities of all proteases were stopped by the addition of 1% [v/v] formic acid (FA). The pH of each sample was controlled and adjusted to <3.

Peptides were cleaned directly via solid-phase extraction on SepPak Vac 1 cc (50 mg) tC18 cartridges (Waters, Eschborn, Germany). Cartridges were wetted with 1 mL 100% acetonitrile and 1 mL 0.1% [v/v] FA in 50% [v/v] acetonitrile. Cartridge equilibration was performed by adding 2 × 1 mL of 0.1% FA [v/v] in H_2_O. Acidified peptides (pH < 3) were loaded onto the cartridges and washed two times with 0.1% FA [v/v] in H_2_O and eluted 2× in 200 *µ*L of 0.1% FA [v/v] in 50% [v/v] acetonitrile. Cleaned peptides were dried in a vacuum centrifuge and stored at −20 °C. The final peptide concentration was determined with the Pierce peptide quantification kit (Thermo Fisher Scientific, Bremen, Germany), according to the manufacturers’ instructions.

### LC–IMS–MS/MS analysis

A nanoElute HPLC (Bruker Daltonics, Bremen, Germany) was coupled to a timsTOF Pro ion-mobility spectrometry quadrupole time of flight mass spectrometer (Bruker Daltonics, Bremen, Germany). Peptides reconstituted in 0.1% [v/v] FA and 200 ng peptides per sample were directly transferred onto an “Aurora” reversed-phase analytical column with an integrated emitter tip (25 cm × 75 *μ*m inner diameter, IonOpticks, Fitzroy, Australia). Solvent A consisted of 0.1% [v/v] FA in LC–MS grade water, solvent B of 0.1% [v/v] FA in 100% acetonitrile (ACN). Peptides were separated on the analytical column at 50 °C via a 70 min gradient at a flow rate of 300 nL min^−1^. A linear gradient from 2% to 37% B for the first 60 min was followed by a 10 min washing step at 95% B.

The timsTOF Pro mass spectrometer was operated in DDA PASEF mode. Automatic recalibration of ion mobility (IM) before each sample run was activated. MS and MS/MS scan range was 100 to 1,700 *m*/*z*, the IM ranges (1/*K*_0_) for the four-range peptide IM fractionation were 0.7 to 0.9, 0.85 to 1.05, 1.0 to 1.2, and 1.15 to 1.35 V s/cm^2^. A polygon filtering was applied in the *m*/*z* and IM area to exclude the low *m*/*z* of singly charged ions for PASEF precursor selection. Ramp and accumulation time was set to 100 ms to achieve close to 100% duty cycle. The number of PASEF ramps was set to 10 with a charge maximum of 5. The quadrupole isolation width was set to 2 for *m*/*z* = 700 and 3 for *m*/*z* = 800. Collision energy was 20 eV for IMy (1/*K*_0_) 0.6 V s/cm^2^ and 59 EV for IM (1/*K*_0_) 1.6 V s/cm^2^, respectively.

### Database search

MaxQuant 2.0.3.0 (Cox and Mann 2008; Prianichnikov et al. 2020) was used to query acquired MS/MS spectra against a modified in-house TAIR10 database (Fuchs et al. 2020) extended with Araport11 entries missing in TAIR10. For all mitochondrial genes, we generated two initial protein entries, one derived from a completely nonedited and one from a completely edited transcript independent from RNA editing efficiency at a given site. Information on RNA editing sites was derived from Bentolila et al. (2013) and Lenz et al. (2018). We then used a Python algorithm to compute permutation variants of each sequence pair to cover closely spaced editing sites independently. To avoid an overwhelming search space, we assumed that changes with large distances occur on different peptides: editing sites in close proximity are grouped, and permutations are computed separately for each group. This strategy ensures that we have all possible peptides in our database, even if we do not have all permutations for a given protein. In total, our database contains 70,586 protein entries. Carbamidomethyl (C) was specified as a fixed modification, oxidation (M) and acetylation (protein N-Term) were considered as variable modifications. Default parameters were used with the following exceptions: calculation of iBAQ values was activated, and the options “Log fit” and “charge normalization” were enabled. For Trypsin digests, Trypsin/P was specified as the proteolytic enzyme with a maximum of two missed cleavages. For Lys-C digests, LysC/P was specified as the proteolytic enzyme with twp maximum missed cleavages. For chymotrypsin digests, chymotrypsin+ was specified as the proteolytic enzyme with a maximum of four missed cleavages. For Asp-N digests, AspN and GluN were set as proteolytic enzymes with three missed cleavage sites. For Glu-C digests, Glu-C and AspC were specified as the proteolytic enzymes with a maximum of three missed cleavages. For Arg-C digests, ArgC and LysC/P were set as proteolytic enzymes with a maximum of two missed cleavages. For all digests, the minimal peptide length was set to seven amino acids, and the maximum peptide mass to 4,600 Da. Identification transfer between individual runs via the “Match between runs” feature was enabled for LC–MS files of identical digest and TIMS fraction. The match time window was set to 0.7 min, and the alignment time window was set to 20 min. The match IM window was set to 0.05, and alignment IM was set to 1. For all digests, the FDR was 1% at both the PSM and protein levels.

### Manual inspection of nonedited peptides

For the analysis of partial RNA editing, we only considered peptides with an Andromeda peptide score of at least 40. To consider peptides, we additionally required the amino acid position affected by RNA editing to be covered by either a *b-ion* or a *y-ion* in the MS/MS spectra. We only considered peptides identified in at least two distinct MS/MS spectra to avoid misinterpretation based on “one-hit wonders”. For each amino acid exchange resulting from partial editing, we checked whether a PTM at the edited amino acid could cause a similar mass gain or loss. For this, we determined the mass change caused by the amino acid exchange and then searched for PTMs with similar mass (±2 Da) on the UNIMOD platform (Creasy and Cottrell 2004). For none of the partial editing events described in this study we could identify a PTM with similar mass at the edited amino acid. PTMs with an even larger mass deviation would have been easily identified by the mass spectrometers used here, and corresponding spectra would not have been assigned to the nonedited peptide sequences.

### Protein quantification

To quantify proteins across samples and digests, we used the iBAQ strategy according to Schwanhäusser et al. (2011): Protein intensity is calculated as the sum of all identified peptide intensities, divided by the number of theoretically observable peptides, calculated by in silico protein digestion. For calculating protein quantities, we only considered protein groups that had iBAQ values of >0 in three out of three biological replicates in at least one digest (4,692 protein groups). iBAQ values were normalized by dividing them by the sum of all iBAQs in a sample and subsequently averaged between all three replicates of each digest, giving the protein quantity in each of the six digests. To calculate the protein quantity across all digests, we averaged the calculated protein quantities of the single digests and only considered those with a value >0 since not all proteins are covered by different proteases. By combining the quantitative information of all six digests, instead of only using the information from one digest, we minimize protease bias, which could distort the representation of proteins within a proteome, since different protease specificities can lead to over- or under-representation of digested proteins (Peng et al. 2012; Tsiatsiani and Heck 2015).

## Supplementary Material

kiad655_Supplementary_Data

## Data Availability

The mass spectrometry proteomics data as well the complete MaxQuant analysis output have been deposited to the ProteomeXchange Consortium (http://proteomecentral.proteomexchange.org) via the Mass Spectrometry Interactive Virtual Environment (MassIVE) repository with the dataset identifier MSV000092578, which can be accessed via the following link: https://massive.ucsd.edu/ProteoSAFe/dataset.jsp?accession=MSV000092578. Data can be inspected in detail by the built-in viewer function of MaxQuant. A compatible version of MaxQuant has been provided with the data. A documentation on how to start and use the viewer with the dataset can be found here: https://www.maxquant.org. The complete “deep mitochondrial proteome” dataset can be interactively explored here: https://proteomeexplorer.de/2/. Annotated spectra for all results can be viewed using MS-Viewer (Baker and Chalkley 2014) at https://msviewer.ucsf.edu/prospector/cgi-bin/mssearch.cgi? report_title=MS-Viewer&search_key=b097d5ot45&search_name=msviewer.

## Supplementary data

**Supplementary Figure S1.** Summary of peptide and protein identifications across the six different digests and replicates.

**Supplementary Figure S2.** Comparison of proteome coverage of the current study and the study presented by Fuchs et al. (2020).

**Supplementary Figure S3.** Representative MS/MS spectra of peptides specific for RNA editing site atp4eU416TIp98.

**Supplementary Figure S4.** Representative MS/MS spectra of peptides specific for RNA editing site rpl5eU121PSp3.

**Supplementary Figure S5.** Representative MS/MS spectrum of a peptide specific for RNA editing sites rps3eU1571AVp97, rps3eU1580SFp11, and rps3eU1598SLp97.

**Supplementary Figure S6.** Representative MS/MS spectra of peptides specific for RNA editing site rps3eU887SLp73.

**Supplementary Figure S7.** Representative MS/MS spectra of peptides specific for RNA editing site atp4eU416TIp98.

**Supplementary Figure S8.** Representative MS/MS spectra of peptides specific for RNA editing site rps3eU887SLp73.

**Supplementary Figure S9.** Representative MS/MS spectra of peptides specific for RNA editing site rps3eU887SLp73.

**Supplementary Figure S10.** Representative MS/MS spectra of peptides specific for RNA editing sites rps3eU1571AVp97 and rps3eU1580SFp11.

**Supplementary Figure S11.** Representative MS/MS spectra of peptides indicative of AS events of AT3G20000 transcripts.

**Supplementary Figure S12.** Representative MS/MS spectra of peptides indicative of AS events of AT4G26910 transcripts.

**Supplementary Table S1.** Overview of all peptides identified in this study.

**Supplementary Table S2.** Overview of all proteins identified in this study.

**Supplementary Table S3.** Overview of RNA editing sites of mitochondrial mRNA and their detected editing state on protein level.

**Supplementary Table S4.** Overview of all potentially alternative-spliced proteins in our data set.

**Supplementary Data Set 1.** Atomic model of the small mitoribosomal subunit as presented in Fig. 10.

## Dive Curated Terms

The following phenotypic, genotypic, and functional terms are of significance to the work described in this paper:

ATP CHEBI: CHEBI:15422

ATP4 Gramene: ATMG00640
ATP4 Araport: ATMG00640

peptides CHEBI: CHEBI:16670

proteins CHEBI: CHEBI:36080

EDTA CHEBI: CHEBI:4735

TOM40 Gramene: AT3G20000
TOM40 Araport: AT3G20000

OGDH Gramene: AT4G26910
OGDH Araport: AT4G26910
