# Supplementary material for: Deep proteomics reveals incorporation of unedited proteins into mitochondrial protein complexes in Arabidopsis
Source: Plant Physiol. 2023 Dec 7;195(2):1180–99. doi: 10.1093/plphys/kiad655 (PMC11142381; doi:10.1093/plphys/kiad655)
Supplement: kiad655_Supplementary_Data [file kiad655_supplementary_data.zip › PP2023RA01285R1Merged_Decision_Summary_PDF.pdf]

Plant Physiology®

Proteomic Exploration of Arabidopsis mitochondria reveals incorporation of non-edited proteins into protein complexes

Nils Rugen, Michael Senkler, and Hans-Peter Braun

Review Timeline:

Submission Date:	22-Aug-2023
Editorial Decision:	23-Sep-2023
Revision Received:	26-Oct-2023
Accepted:	12-Nov-2023

23-Sep-2023

Dr. Nils Rugen
Leibniz Universitat Hannover Institut fur Pflanzengenetik
Hannover
Germany

RE: Proteomic Exploration of Arabidopsis mitochondria reveals incorporation of non-edited proteins into protein complexes

Dear Nils,

We have received reviews of your manuscript, which you may view at the end of this letter. On the basis of the advice received and my own evaluation, I believe that, pending revision, this article may ultimately be acceptable for publication in Plant Physiology.

I have been lucky to obtain reports from three experts in the fields of plant mitochondrial gene expression and proteomics. All reviewers agree that the dataset you report on is technically of highest quality and will make for a valuable resource. Yet, there are justified suggestions for improvement of presentation and the concern that more effort could have been spent on working out the biological insights. No additional experimentation will be required, but a thorough revision of the Discussion and the Figures. In particularly the key biological finding of proteins from unedited transcripts should draw on the full body of evidence available. I believe the reviews themself are instructive and provide excellent guidance for the revision.

When you are ready to submit your revision, click the below link and find the red arrow under your Author Tasks heading for this revision submission.

If you want to visit this version of the manuscript, you may click the link below and then click on the folder that reads "Post Decision Manuscripts".

Link Not Available

When uploading the revised version of this article, please be sure to include as complete an answer as possible to the referees' criticisms during the first round of reviews in the "response to reviewers" section.

Plant Physiology considers a revision for up to 60 days after a manuscript has been provisionally accepted; limited extension of this timeframe is at the discretion of the Monitoring Editor. A reminder notice will be sent to you 2 weeks before the deadline, at which time you may choose to contact the editorial office to request an extension; if so, please provide a justification for your request. After the expiration of this deadline, the article would need to be submitted as a resubmission and would be handled as such.

Thank you very much for giving us an opportunity to review this work. I look forward to receiving the next version.

Sincerely,
Plant Physiology Board of Editors

Want to add this revision deadline to your calendar? Click below!

-- Reviewer comments:
Reviewer #1 (Comments for the Author):

Review to PP2023-RA-01285
RNA-editing is a key regulatory step in plant organellar gene expression, where (mostly) numerous C-to-U replacements affect the coding sequences of many of the mitochondrial and plastid transcripts in angiosperms. PPR proteins were found to a have

pivotal roles in this essential step in plant organellar gene expression, in which they provide both the binding septicity as well as the deamination activitoes . It is apparent that while some of the RNA editing sites are "fully edited", the splicing efficiencies of various other loci may vary, or even regulated at specific tissues and under developmental or environmental signals. The MS by Nils Rugena et al, provides a comprehensive investigation into the mitochondrial proteome of Arabidopsis thaliana (with about 4,700 proteins identified and studied), in which the authors looked into the protein sequences that are translated from edited, partially edited and non-edited transcripts of mitochondria and chloroplasts. This involved the use of multiple endoproteases, in order to increase the coverage of the proteins. The dataset led to the identification of many proteins that are assigned to the mitochondria, including editing and alternative splicing varients. The authors show that the majority of mitochondrially-encoded proteins are derived from edited mRNAs, further indicating that a complete maturation of an organellar transcripts is a prerequisite for its efficient translation. Yet, proteins translated from some non-edited transcripts corresponding to mitoribosomes and into the ATP synthase complex, a molecular mechanism that may indicate that RNA editing can increase the organellar proteomes from a given set of gene loci, a phenomenon of great importance to the field of organellar genome expression, RNA metabolism and protein analyses.
I find that the authors have produce a comprehensive dataset in the molecular, biochemical and genetic levels to address the molecular basis of RNA editing and the translation of mtRNAs in plant mitochondria, and that it brings with important and novel insights into these important steps in gene expression. The authors have ample experience in mitochondria biochemistry and proteomic analyses. Furthermore, the Proteome Explorer database they produced is a very useful tool for mitochondria research in both plant and not-plant systems. The work, figures and the data organization are of high quality, and I only have minor comments.

Specific comments:
1. P. 5 3rd paragraph. The authors may reconsider the term "aggregated proteome data".
2. Fig. 10. Change the wording in RPS3 into 'other mitoribosomal proteins"
3. As indicated by Fig. 10 the authors may consider a similar representation for ATP synthase and ATP4 (Peripheral stalk subunit b).
4. One thing that I find to be missing from the discussion is the fact that such proteome variations supported by the authors in the case of Atp4 and RPS3 may be related with physiological or environmental signals. A follow-up of the study may include different plant organs and plants grown under sub-optimal growth conditions.
5. The authors need to explain where the specificity for the translation comes from. One idea/assumption is that the PPRs or editosome while they're sitting on the transcript until editing is complete, physically block translation. Please comment on more clearly.

Reviewer #2 (Comments for the Author):

The manuscript "Proteomic Exploration of Arabidopsis mitochondria reveals incorporation of non-edited proteins into protein complexes " by Nils Rugen, Michael Senkler & Hans-Peter Braun is a type of manuscript that will be enthusiastically received by protein scientists, but the average "Plant Physiology" journal reader looks for less technical, more biologically oriented information. In this context, the finding that incompletely edited proteins were identified in mitochondrial complexes is important and fascinating. However, this aspect has not been deeply explored except for checking the location of unedited peptides in the protein structure. . On the other hand, Proteome Explorer, a new public online platform recommended in this manuscript, will be very useful for people identifying and characterizing proteins. Perhaps it would be better if most of the data on the improved shotgun technique were published in a proteomics journal, while the data on the unedited proteins present in protein complexes (shotgun + complex profiling data) could be published as a short report/news in a journal intended for the general public.
Major revison:
1.It is worth adding a brief discussion of the amount/extent of unedited proteins in mitochondrial complexes. The most important questions: Is this a random or quite control phenomenon? Is there a correlation with transcript editing efficiency, or is there a correlation with the number of unedited transcripts associated with ribosome (Ribo-seq data)? For comparison, it will also be profitable to use data from complex profiling performed by others. Ideally, the effect of reduced protein editing efficiency (respective mutant) on the level of unedited proteins in the complex and the complex's functionality should be tested experimentally
2.In the chapter describing the detection of peptides corresponding to unedited transcripts the authors do not comment at all on the fact that more unedited peptides were found by complexome profiling than deep shotgun (Table 3: 8 vs. 6). Furthermore, what is the interpretation that for several proteins only unedited peptides but no edited ones were found (e.g., rps3eU187LFp2 or ccmFceU925PSp2) in the complexome profiling data.
Minor revison:
1. Title: Reading the title gives the impression that the process of incorporating incompletely edited proteins into protein complexes is quite common, Was this the authors' intention? In fact only a few unedited peptides were reported in this study.
2. Abstract: Two sentences from the abstract express almost the same idea: 1. To obtain maximum coverage of the proteome, proteins were digested in parallel with six different endoproteases. 2. Thanks to the use of many endoproteases, the coverage of proteins with identified peptides was particularly high.

3. Results and discussion: Generally, the text should be condensed, and some methodological details transferred to methods.
4. Page 6; lines 13-21 - This section of text is unclear in terms of percentages; for example, how can chloroplast proteins account for 210 %?
5. Page 8: It would be better to reword the second section, which begins with the words "We only considered peptides with an Andromeda.....", to make the description more understandable to the average reader.
6. Description of Figure 7: heat maps of clustered proteins belonging to the small mitoribosomal subunit, not the large mitoribosomal subunit.

Reviewer #3 (Comments for the Author):

Rugen et al has undertaken a very detailed MS/MS analysis of mitochondrial enriched extracts from Arabidopsis cell culture using 6 different proteolytic enzymes, fractionation and TIMS TOF Pro analysis to provide the largest study yet, in terms of peptide coverage, of the proteome of plant mitochondria. The authors have made a nice interactive database (proteomeexplorer.de) that allows a user to observe the peptides found after different enzyme digests. This both showcases the value of multiple enzymes in Arabidopsis proteomics for more coverage and is useful for readers with specific interest in targeted MS for a particular protein to select targets from this resource. I have used it already!
The approach taken at analysis of the set as a whole has not tried to expand the mitochondrial proteome per se, different degrees of mitochondrial enrichment or a further refinement in mitochondrial purity have not been sought. Instead, the authors have used a largely agreed standard mitochondrial isolation technique and a set of agreed mitochondrial proteins from past research and looked at the larger set of peptides that match to them to explore three questions:

1. Is there experimental evidence for unedited proteins arising from the plant mitochondrial genome? Answer - yes, some.
The authors provide a small number of cases where peptide MS/MS are found that appear by mass and MS/MS fragmentation to match to the unedited versions of mitochondrial encoded proteins. In some cases they show that not only can these be found in cell culture mitochondria (is it possible more sloppy protein quality control may be carried out in cell culture?) but a number of them can also be found in leaf mitochondria and in native complexes consistent with incorporation of unedited proteins into mature native protein complexes of mitochondria. Overall this is a compelling experiment case and it is new evidence. To my knowledge such data has not previously been presented and will be of interest to the RNA editing community. However, I think the authors should acknowledge and deal with a few likely queries from readers. (A) The MS analysis methods used are not an exhaustive analysis of PTMs and typical mass losses of peptides that could arise by post-translational processes. The authors need to clarify in detail that the loss or gain of mass associated with the claim of lack of editing could not reasonably be derived from low stoichiometry PTMs of the edited amino acid residues in each case. I think this is largely a case of taking each in turn, looking at mass differences and using the exhaustive literature on PTMs to make the case for lack of editing over potential PTM mass gain or mass loss. (B) How abundant are these variants in reality and how could they have an impact on biological function if they represent a loss of function but only account for <1% of the protein of interest in mitochondria? This is really a question of it they represent loss of function or gain of function effects in the low stoichiometry version, this should be discussed.

2. Is there evidence of alternative splice variants for nuclear encoded proteins? Answer - Yes a few.
In these cases, changes to the N-termini of proteins are noted, however, the consequences of this for mitochondrial targeting of proteins has not been discussed by the authors. There is no real discussion about the expression of these different alternative splice variants and thus if any transcriptional signature might be a factor in their relative importance. Multiple resources in recent years have provided AS specific transcript data in different tissue types and development responses in Arabidopsis. Is there any evidence for differential expression of the alternative spliced proteins found here that might given a biological value to their identification?

3. How abundant are different mitochondrial proteins? A range of approaches other than IBAQ have been developed to predict absolute abundance. So how consistent are they for a large dataset like this with good coverage? Lack of good peptide coverage for a particular protein is often pointed out as one of the problems with these types of estimations. Is IBAQ really the best? This appears to be a good dataset to assess this issue, but instead only IBAQ is considered in prediction of protein abundance. This seems like a missed opportunity given this same approach was also previously used in Fuchs et al.

Overall I think the figures could be improved:
Figure 1 is like a powerpoint slide from a general talk - it should include a range of the numbers from the results of the manuscript along the process to show what is gained in my opinion and therefore will be more generally useful as an illustration of the paper.
Figure 2 does not incorporate any variation data between the three replicates. As a consequence its not possible to determine if any of the changes in subcellular location assignment between enzyme digestions mean anything statistically, so its not clear what the figure is illustrating.
Figure 3 does not well illustrate the issue it aims to address. I suggest some combination of log10 protein abundance with different subcellular assignments so that it can be seen how the mitochondrial proteome overlaps and interleaves with other subcellular contaminants or interactors.
Figure 5 loses any context of the coverage of specific proteins between study groups. Really that issue is more interesting than

what is shown. I would urge the authors to think of a more sophisticated way to illustrate this.
Figure 7 - why is IAFFVESLTSEK (blue in B (i)) peaking between 0.76 and 0.33 MDa when other ribosome proteins don't? the same is true for IDYAPVEVSTR. Perhaps the heatmap in A is not well representing the peptide intensities shown in B? it would be helpful to show other representative rps3 edited peptides, or a consensus peptide intensity for all peptides for rps3?

Supp figures - it would be more helpful if the x-axis for comparable MS/MS spectra are given in the same range - different ranges make comparisons and orientation very hard for readers.

Minor: methods: use of ug rather than µg in some parts needs to be fixed.

Response to the reviewers

Response to reviewer #1

Reviewer #1: RNA-editing is a key regulatory step in plant organellar gene expression, where (mostly) numerous C-to-U replacements affect the coding sequences of many of the mitochondrial and plastid transcripts in angiosperms. PPR proteins were found to a have pivotal roles in this essential step in plant organellar gene expression, in which they provide both the binding septicity as well as the deamination activitoes . It is apparent that while some of the RNA editing sites are "fully edited", the splicing efficiencies of various other loci may vary, or even regulated at specific tissues and under developmental or environmental signals. The MS by Nils Rugena et al, provides a comprehensive investigation into the mitochondrial proteome of Arabidopsis thaliana (with about 4,700 proteins identified and studied), in which the authors looked into the protein sequences that are translated from edited, partially edited and non-edited transcripts of mitochondria and chloroplasts. This involved the use of multiple endoproteases, in order to increase the coverage of the proteins. The dataset led to the identification of many proteins that are assigned to the mitochondria, including editing and alternative splicing varients. The authors show that the majority of mitochondrially-encoded proteins are derived from edited mRNAs, further indicating that a complete maturation of an organellar transcripts is a prerequisite for its efficient translation. Yet, proteins translated from some non-edited transcripts corresponding to mitoribosomes and into the ATP synthase complex, a molecular mechanism that may indicate that RNA editing can increase the organellar proteomes from a given set of gene loci, a phenomenon of great importance to the field of organellar genome expression, RNA metabolism and protein analyses.
I find that the authors have produce a comprehensive dataset in the molecular, biochemical and genetic levels to address the molecular basis of RNA editing and the translation of mtRNAs in plant mitochondria, and that it brings with important and novel insights into these important steps in gene expression. The authors have ample experience in mitochondria biochemistry and proteomic analyses. Furthermore, the Proteome Explorer database they produced is a very useful tool for mitochondria research in both plant and not-plant systems. The work, figures and the data organization are of high quality, and I only have minor comments.

Our response: Thank you, the feedback is highly appreciated!

Specific comments:

Reviewer #1: P. 5 3rd paragraph. The authors may reconsider the term "aggregated proteome data".

Our response: we changed the sentence into “Altogether, our proteome dataset comprises more than 100,000 distinct peptides…”

Reviewer #1: Fig. 10. Change the wording in RPS3 into 'other mitoribosomal proteins"

Our response: Thank you, we corrected the figure.

Reviewer #1: As indicated by Fig. 10 the authors may consider a similar representation for ATP synthase and ATP4 (Peripheral stalk subunit b).

Our response: The structure of the mitochondrial ATP synthase complex has not been resolved in plants (no atomic model available). Based on the ATP synthase structure from yeast we can only estimate where the editing site probably is located, as discussed in the conclusion and outlook section. We therefore are in favor of not adding a figure on this issue.

Reviewer #1: One thing that I find to be missing from the discussion is the fact that such proteome variations supported by the authors in the case of Atp4 and RPS3 may be related with physiological or environmental signals. A follow-up of the study may include different plant organs and plants grown under sub-optimal growth conditions.

Our response: We added a paragraph to the conclusion and outlook section covering possible biological reasons for partial editing and suggestions on future experiments for investigating this issue.

Reviewer #1: The authors need to explain where the specificity for the translation comes from. One idea/assumption is that the PPRs or editosome while they're sitting on the transcript until editing is complete, physically block translation. Please comment on more clearly.

Our response: In the introduction, we now mention the post- or co-translational processes that might be responsible for translating primarily fully edited transcripts.

Reviewer #2 (Comments for the Author):

The manuscript "Proteomic Exploration of Arabidopsis mitochondria reveals incorporation of non-edited proteins into protein complexes " by Nils Rugen, Michael Senkler & Hans-Peter Braun is a type of manuscript that will be enthusiastically received by protein scientists, but the average "Plant Physiology" journal reader looks for less technical, more biologically oriented information. In this context, the finding that incompletely edited proteins were identified in mitochondrial complexes is important and fascinating. However, this aspect has not been deeply explored except for checking the location of unedited peptides in the protein structure. On the other hand, Proteome Explorer, a new public online platform recommended in this manuscript, will be very useful for people identifying and characterizing proteins. Perhaps it would be better if most of the data on the improved shotgun technique were published in a proteomics journal, while the data on the unedited proteins present in protein complexes (shotgun + complex profiling data) could be published as a short report/news in a journal intended for the general public.

Major revison:

1. It is worth adding a brief discussion of the amount/extent of unedited proteins in mitochondrial complexes. The most important questions: Is this a random or quite control phenomenon? Is there a correlation with transcript editing efficiency, or is there a correlation with the number of unedited transcripts associated with ribosome (Ribo-seq data)?

   Our response: Percentage of non-edited peptides now is given for ATP4 and RPS3 based on measured peptide intensity. While the amount of the non-edited peptide of ATP4 within the native ATP synthase complex is <1%, the amount of the non/partially edited peptides in RPS3 is in the range of 4-9%. This is now given in the corresponding section of the results part. The editing sites at rps3eU1571AVp97 and rps3eU1580SFp11 are not covered by the RiboSeq approach of Planchard et al. 2018. The amount of non-edited molecules (transcripts or peptides derived from non-edited transcripts) varies on the transcript and the peptide level, as now discussed.

2. For comparison, it will also be profitable to use data from complex profiling performed by others. Ideally, the effect of reduced protein editing efficiency (respective mutant) on the level of unedited proteins in the complex and the complex's functionality should be tested experimentally

   Our response: Complexome profiling data usually are performed using an one-dimensional lane of a blue-native (BN) gel, which is dissected into several small pieces from bottom to top, all of which are analyzed by label-free quantitative shotgun proteomics. In normal BN-PAGE experiments, the ribosomes do not show up due to the covered molecular mass range. Only by using large-pore (lp) BN PAGE analyses, the ribosomal subunits can be detected. To our knowledge, this only has been performed by Rugen et al. 2019 and Rugen et al. 2022. These data have been used for our data evaluation.
   We agree, the functionality of complexes including non-edited peptides has to be addressed experimentally. This is now mentioned in our conclusion and outlook section.

3. In the chapter describing the detection of peptides corresponding to unedited transcripts the authors do not comment at all on the fact that more unedited peptides were found by complexome profiling than deep shotgun (Table 3: 8 vs. 6).

   Our response: The complexome profiling fractions are by far less complex than the total mitochondrial fractions analyzed in our deep-shotgun experiment (about 500 versus about 5000 proteins). In fact, specific peptides usually are much enriched in their peak complexome profiling factions. As a consequence, detection of low-abundant non-edited peptides is facilitated in complexome profiling fractions.

4. Furthermore, what is the interpretation that for several proteins only unedited peptides but no edited ones were found (e.g., rps3eU187LFp2 or ccmFceU925PSp2) in the complexome profiling data.

   Our response: We have no interpretation. Indeed, for the sites mentioned, which have a low editing frequency on the transcript level, we only detected the non-edited peptides, but not any peptides derived from the edited transcript. We assume that this is due to the low percentage of edited transcripts.

Minor revison:

1. **Title**: Reading the title gives the impression that the process of incorporating incompletely edited proteins into protein complexes is quite common, Was this the authors' intention? In fact only a few unedited peptides were reported in this study.

   Our response: We reflected about changing the title, but would like to keep the title as it stands. Indeed, most peptides detectable in the Arabidopsis mitochondrial proteome correspond to fully edited transcripts. However, this is not in contradiction with our title.

2. **Abstract**: Two sentences from the abstract express almost the same idea:
   a. To obtain maximum coverage of the proteome, proteins were digested in parallel with six different endoproteases.
   b. Thanks to the use of many endoproteases, the coverage of proteins with identified peptides was particularly high.

   Our response: Thank you for pointing out this issue; we changed the second sentence to "The coverage of proteins by identified peptides was particularly high in comparison to single-protease digests."

3. Results and discussion: Generally, the text should be condensed, and some methodological details transferred to methods.

   Our response: Details on interpreting the MS spectra have been transferred to the Experimental procedures section.

4. Page 6; lines 13-21 - This section of text is unclear in terms of percentages; for example, how can chloroplast proteins account for 210 %?

Our response: Thank you for this comment; we corrected this typing error. The number of plastidic proteins (according to SUBA5) accounts for 21% of all identified proteins. The abundance (based in iBAQ) of each individual protein is not yet considered here.

5. Page 8: It would be better to reword the second section, which begins with the words "We only considered peptides with an Andromeda.....", to make the description more understandable to the average reader.

   Our response: We agree that this paragraph is indeed very technical and not necessary for the reader to follow our analysis. We moved this paragraph to the “Experimental Procedures” section, entitled by the subheading “Manual Inspection of non-edited peptides”. The results section now includes the following statement: “To ensure correct identification of non-edited peptides, they had to fulfill certain criteria, which are given in the ‘Experimental Procedures’ section.”

6. Description of Figure 7: heat maps of clustered proteins belonging to the small mitoribosomal subunit, not the large mitoribosomal subunit.

   Our response: we corrected the figure description.

Reviewer #3 (Comments for the Author):

Rugen et al has undertaken a very detailed MS/MS analysis of mitochondrial enriched extracts from Arabidopsis cell culture using 6 different proteolytic enzymes, fractionation and TIMS TOF Pro analysis to provide the largest study yet, in terms of peptide coverage, of the proteome of plant mitochondria. The authors have made a nice interactive database (proteomeexplorer.de) that allows a user to observe the peptides found after different enzyme digests. This both showcases the value of multiple enzymes in Arabidopsis proteomics for more coverage and is useful for readers with specific interest in targeted MS for a particular protein to select targets from this resource. I have used it already!
The approach taken at analysis of the set as a whole has not tried to expand the mitochondrial proteome per se, different degrees of mitochondrial enrichment or a further refinement in mitochondrial purity have not been sought. Instead, the authors have used a largely agreed standard mitochondrial isolation technique and a set of agreed mitochondrial proteins from past research and looked at the larger set of peptides that match to them to explore three questions:

1. Is there experimental evidence for unedited proteins arising from the plant mitochondrial genome? Answer - yes, some.
   The authors provide a small number of cases where peptide MS/MS are found that appear by mass and MS/MS fragmentation to match to the unedited versions of mitochondrial encoded proteins. In some cases they show that not only can these be found in cell culture mitochondria (is it possible more sloppy protein quality control may be carried out in cell culture?) but a number of them can also be found in leaf mitochondria and in native complexes consistent with incorporation of unedited proteins into mature native protein complexes of mitochondria. Overall this is a compelling experiment case and it is new evidence. To my knowledge such data has not previously been presented and will be of interest to the RNA editing community. However, I think the authors should acknowledge and deal with a few likely queries from readers.
   a. The MS analysis methods used are not an exhaustive analysis of PTMs and typical mass losses of peptides that could arise by post-translational processes. The authors need to clarify in detail that the loss or gain of mass associated with the claim of lack of editing could not reasonably be derived from low stoichiometry PTMs of the edited amino acid residues in each case. I think this is largely a case of taking each in turn, looking at mass differences and using the exhaustive literature on PTMs to make the case for lack of editing over potential PTM mass gain or mass loss.

      Our response: Thank you for bringing up this point. We now manually evaluated all peptides derived from differentially edited transcript sites and conclude that no known PTMs can account for the observed differences in molecular mass. Details are given in the Experimental Procedures section in a new paragraph entitled “Manual Inspection of non-edited Peptides“.

   b. How abundant are these variants in reality and how could they have an impact on biological function if they represent a loss of function but only account for <1% of the protein of interest in mitochondria? This is really a question of it they represent loss of function or gain of function effects in the low stoichiometry version, this should be discussed.

Our response: In the new manuscript version, we make estimates of the abundances of the differential peptides covering editing-sites and discuss consequences of their incorporation into protein complexes. For an absolute quantification of edited and non-edited proteins, one would need to use isotopically labeled spike-in standards for each peptide pair representing a RNA editing site.

2. Is there evidence of alternative splice variants for nuclear encoded proteins? Answer - Yes a few. In these cases, changes to the N-termini of proteins are noted, however, the consequences of this for mitochondrial targeting of proteins has not been discussed by the authors. There is no real discussion about the expression of these different alternative splice variants and thus if any transcriptional signature might be a factor in their relative importance. Multiple resources in recent years have provided AS specific transcript data in different tissue types and development responses in Arabidopsis. Is there any evidence for differential expression of the alternative spliced proteins found here that might given a biological value to their identification?

   Our response: We evaluated the predicted targeting information of the splice variants. In case of TOM40, both splice variants are mainly assigned to the mitochondria. In the case of OGDC-E2, the shorter variant is not predicted to be transported to the mitochondria. This information has been added to our manuscript. We evaluated transcriptome datasets containing information of transcript abundance in different tissues and at different plant cultivation conditions but could not find any new information on the biological implication of the described splice variants.

3. How abundant are different mitochondrial proteins? A range of approaches other than IBAQ have been developed to predict absolute abundance. So how consistent are they for a large dataset like this with good coverage? Lack of good peptide coverage for a particular protein is often pointed out as one of the problems with these types of estimations. Is IBAQ really the best? This appears to be a good dataset to assess this issue, but instead only IBAQ is considered in prediction of protein abundance. This seems like a missed opportunity given this same approach was also previously used in Fuchs et al.

   Our response: The reliability of IBAQ values correlates with the number of identified peptides per protein. Since the coverage of proteins by identified peptides is especially high in our study, IBAQ values should nicely reflect protein abundance. While we appreciate the idea to use and compare other label-free quantification strategies for shotgun proteome datasets, this goes beyond the aim of our present study. Note: In a recent high-quality multi-protease proteome investigation the same strategy has been used for protein quantification (Sinitcyn et al. 2023; https://www.nature.com/articles/s41587-023-01714-x).

Overall I think the figures could be improved:

Reviewer #3: Figure 1 is like a powerpoint slide from a general talk - it should include a range of the numbers from the results of the manuscript along the process to show what is gained in my opinion and therefore will be more generally useful as an illustration of the paper.

Our answer: Some numbers of our results have been introduced into the figure.

Reviewer #3: Figure 2 does not incorporate any variation data between the three replicates. As a consequence its not possible to determine if any of the changes in subcellular location assignment between enzyme digestions mean anything statistically, so its not clear what the figure is illustrating.

Our answer: We added error bars reflecting the standard deviation of each subcellular localization between replicates. Since this made the stacked bar chart too confusing, we have now used a classic bar chart in which the bars are sorted by the mode of digestion.

Reviewer #3: Figure 3 does not well illustrate the issue it aims to address. I suggest some combination of log10 protein abundance with different subcellular assignments so that it can be seen how the mitochondrial proteome overlaps and interleaves with other subcellular contaminants or interactors.

Our answer: Thank you for this comment. We replaced the Figure by a swarmplot.

Reviewer #3: Figure 5 loses any context of the coverage of specific proteins between study groups. Really that issue is more interesting than what is shown. I would urge the authors to think of a more sophisticated way to illustrate this.

Our answer: We replaced the Figure by a graph illustrating the sequence coverage for each of the detected proteins for our tryptic peptide data (yellow) and combined protease digests, including trypsin (gray). The original figure has been transferred into the Supplementary Material. Furthermore, the sequence coverage by peptides now is indicated for every individual protein in Supp. Table 2.

Reviewer #3: Figure 7 - why is IAFFVESLTSEK (blue in B (i)) peaking between 0.76 and 0.33 MDa when other ribosome proteins don't? the same is true for IDYAPVEVSTR. Perhaps the heatmap in A is not well representing the peptide intensities shown in B? it would be helpful to show other representative rps3 edited peptides, or a consensus peptide intensity for all peptides for rps3?

Our answer: Thank you very much for this comment; we updated the figure. The heat map now shows more clearly that RPS3 indeed peaks in the mass range mentioned and clusters there with some other subunits of the mtSSU. Within the ribosome structure (Waltz. et al. 2020; https://doi.org/10.1038/s41477-020-0631-5), these proteins are located in close proximity to RPS3. We therefore assume that the peak between 0.76 and 0.33 MDa represents a possible assembly intermediate. As suggested by the reviewer, we added an average profile of all RPS3 peptides. We have also reduced the size of the heatmap so that the figure is less complex.

Reviewer #3: Supp figures - it would be more helpful if the x-axis for comparable MS/MS spectra are given in the same range - different ranges make comparisons and orientation very hard for readers.

Our answer: We have adjusted the corresponding figures.

Reviewer #3: Minor: methods: use of ug rather than µg in some parts needs to be fixed.

Our answer: Corrected.

12-Nov-2023
Dr. Nils Rugen
Leibniz Universitat Hannover Institut fur Pflanzengenetik
Hannover
Germany

MSID: PP2023-RA-01285R1
MS TITLE: Proteomic Exploration of Arabidopsis mitochondria reveals incorporation of non-edited proteins into protein complexes

Dear Nils,

I am pleased to inform you that your revised manuscript "Proteomic Exploration of Arabidopsis mitochondria reveals incorporation of non-edited proteins into protein complexes" has been accepted for publication in Plant Physiology. Your manuscript will be checked for consistency with journal formatting and image standards by one of our Science Editors, who will contact you shortly. They may have a number of queries and suggestions for you. Please keep in mind that they work to increase the online visibility of your article and its attraction for readers from the widest of backgrounds. You will have the opportunity to review any revisions, and I hope you will work constructively with them to get the most out of your research. You will also have the opportunity to review all changes when you receive the author proofs for your article.

Once validated for publication, your article will be sent to a Science Editor for a final review of your manuscript and source files. After your final files are uploaded, your article will appear in the next available issue and the online Preview version of your article will be posted after your signed Copyright and Checklist forms and final source files are received.

Please be sure to include the following in your manuscript file:
Harvard style citations
List of all author contributions and funding information at the beginning of manuscript after the titles and author list.
List all supplemental materials associated with the article, with titles, at the end of the ms, before the references and acknowledgments section.
Complete Figure Legends for all figures appearing in the article listed before the Literature Cited.

Thank you and congratulations on your Plant Physiology paper!

Sincerely,

Plant Physiology Board of Editors

=====
FIRST AUTHOR PROFILES
=====

We recognize the hard work that goes into being First Author of a Plant Physiology paper by publishing a "First Author Feature" for each First Author alongside your paper.

If the first author(s) would like to submit information for a feature on Facebook and Twitter, fill out the form by clicking on the link below:

Plant Physiology First Author Feature Form: https://forms.office.com/r/ZBCGZfhm8F
If you cannot access the form through the link above, please contact Rachel at rbelsky@aspb.org.

To upload an image of yourself to accompany your profile, please put your photo in the correct journal folder at the link below. You must label your image: Author First Name Last Name Article Title.

First Author Profile Image Dropbox: https://www.dropbox.com/scl/fo/xz029p7qjr2jml2rp2kmq/h?dl=0&rlkey=6fkxcaqmxx35ap152b2osy1fz

=====
IMPORTANT REMINDER: PEER REVIEW REPORTS
=====

If you opted to publish a peer review report along with your article during the original submission process, it will be prepared by the editorial staff and publicly posted with your manuscript, inside the zip file that contains any other supplemental material. As a reminder, the peer review report is a public record of all comments from editors and reviewers, as well as your prior responses, as you received them in the decision letters for each draft of your manuscript. If you agreed to publish this report and have changed your mind, or are not sure if you selected this option, please contact the editorial office as soon as possible before signing the license agreement from our publisher.

-- Reviewer comments:
Reviewer #1 (Comments for the Author):

The authors have adequately responded to my comments. Although the importance and relevance of the incorporation of non-edited amino acids into the proteome of Arabidopsis mitochondria is awaiting further investigation, the work of Rugen et al indicates in a convincing way that some proteins that are part of the respiratory machinery can be incorporated into their related OXPHOS complexes although are not 'fully matured'. Nevertheless, I find that the data is of high quality and brings with novelty and are of importance to the field of plant organellar biology (as well as to other biological systems). A follow up of this work should be focused on the molecular level and mechanism, i.e., is it a random effect or a controlled/specific process, and whether such effects may be related to an increased proteome (to regulate respiratory functions under various environmental or developmental signals).

Reviewer #3 (Comments for the Author):

The authors have addressed by previous concerns, extended their analysis to confirm editing could not be low stoichiometric PTMs, improved the figures and responded to errors with revisions. This dataset will be of value to plant mitochondrial researchers as an atlas of peptides for future experiments. Given the level of contamination from other organelles it will also support similar work in some of those other locations in Arabidopsis cells. Evidence that a limited amount of unedited RNA derived protein products exist and persist in plant mitochondria opens the opportunity in the future to determine if degrees of editing have a biological significance.
