# Supplementary material for: Deep proteomics reveals incorporation of unedited proteins into mitochondrial protein complexes in Arabidopsis
Source: Plant Physiol. 2023 Dec 7;195(2):1180–99. doi: 10.1093/plphys/kiad655 (PMC11142381; doi:10.1093/plphys/kiad655)
Supplement: kiad655_Supplementary_Data [file kiad655_supplementary_data.zip › Supplemental Data.pdf]

Research Article

Deep Proteomics reveals incorporation of unedited proteins into mitochondrial protein complexes in Arabidopsis

Nils Rugen, Michael Senkler & Hans-Peter Braun

-Supplemental Data-

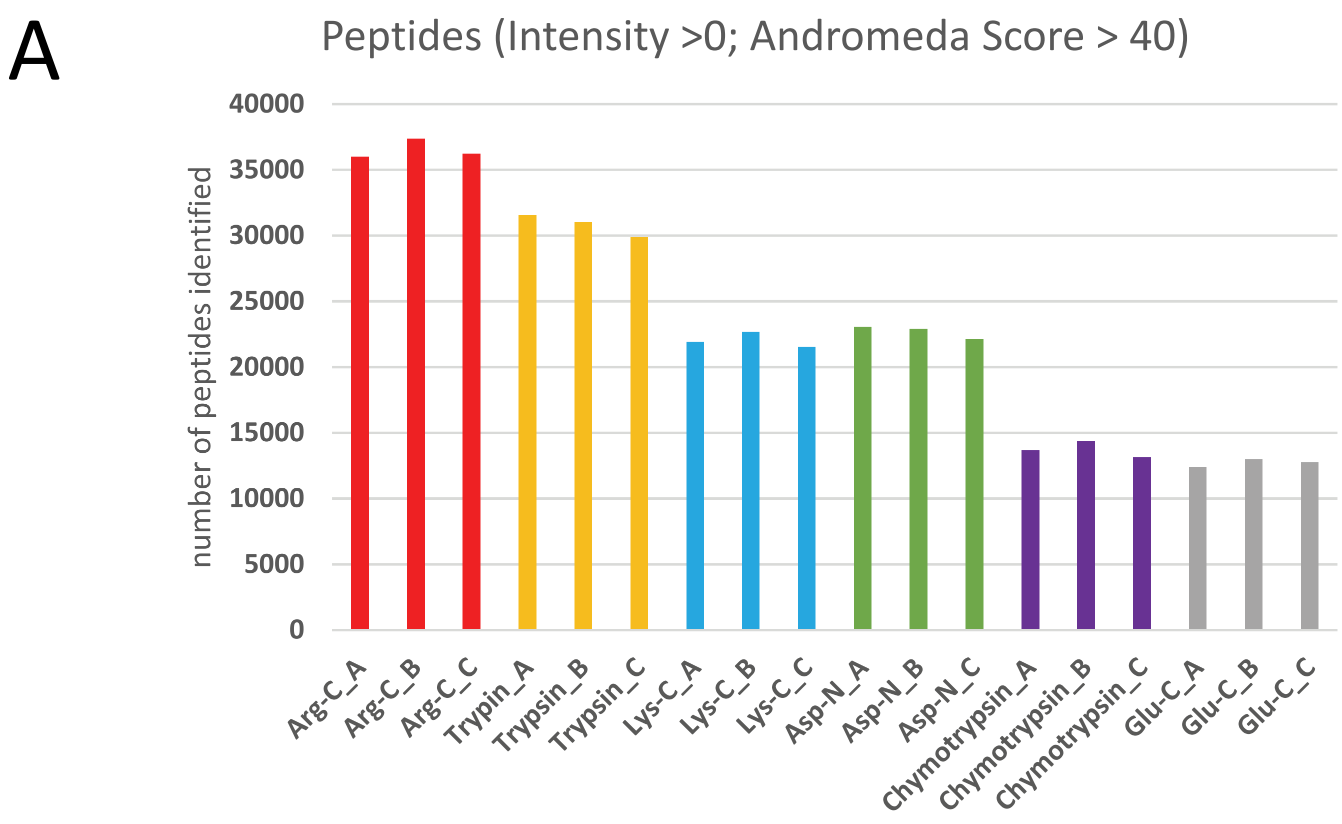

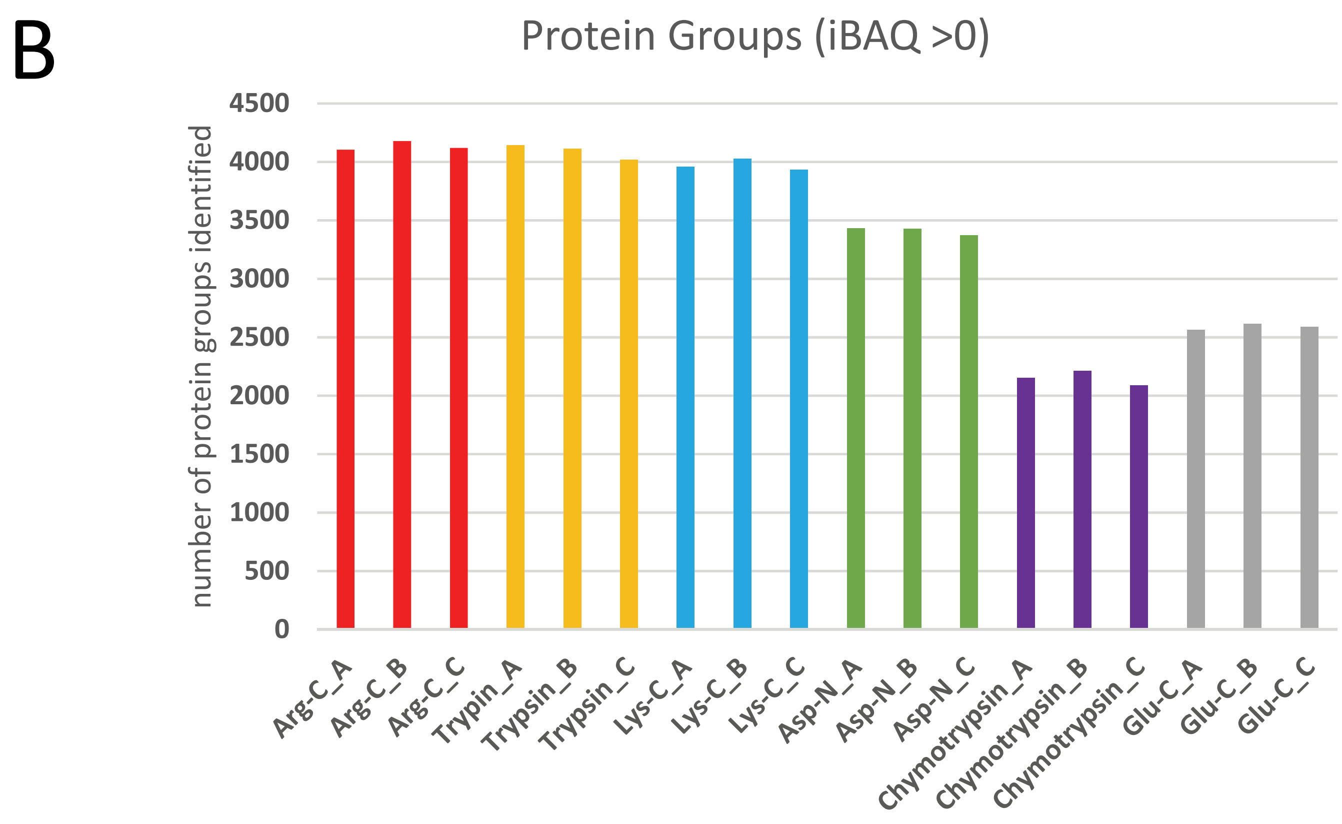

Supplemental Figure S1: Summary of peptide and protein identifications across the six different digests and replicates. All 18 sample are ordered by digest, and replicate. **A:** Overview of identified peptides in the various digests and replicates. A peptide was considered as identified if an intensity >0 was calculated in a given sample and if its Andromeda peptide score was > 40. **B:** Overview of protein identifications in the various digests and replicates. A protein group was considered as identified if an iBAQ value >0 was calculated in a given sample. Red: Arg-C, Yellow: trypsin, Blue: Lys-C, Green: Asp-N, Purple: chymotrypsin, Grey: Glu-C. Lists of all identified peptides and proteins can be found in supplemental tables S1 and S2.

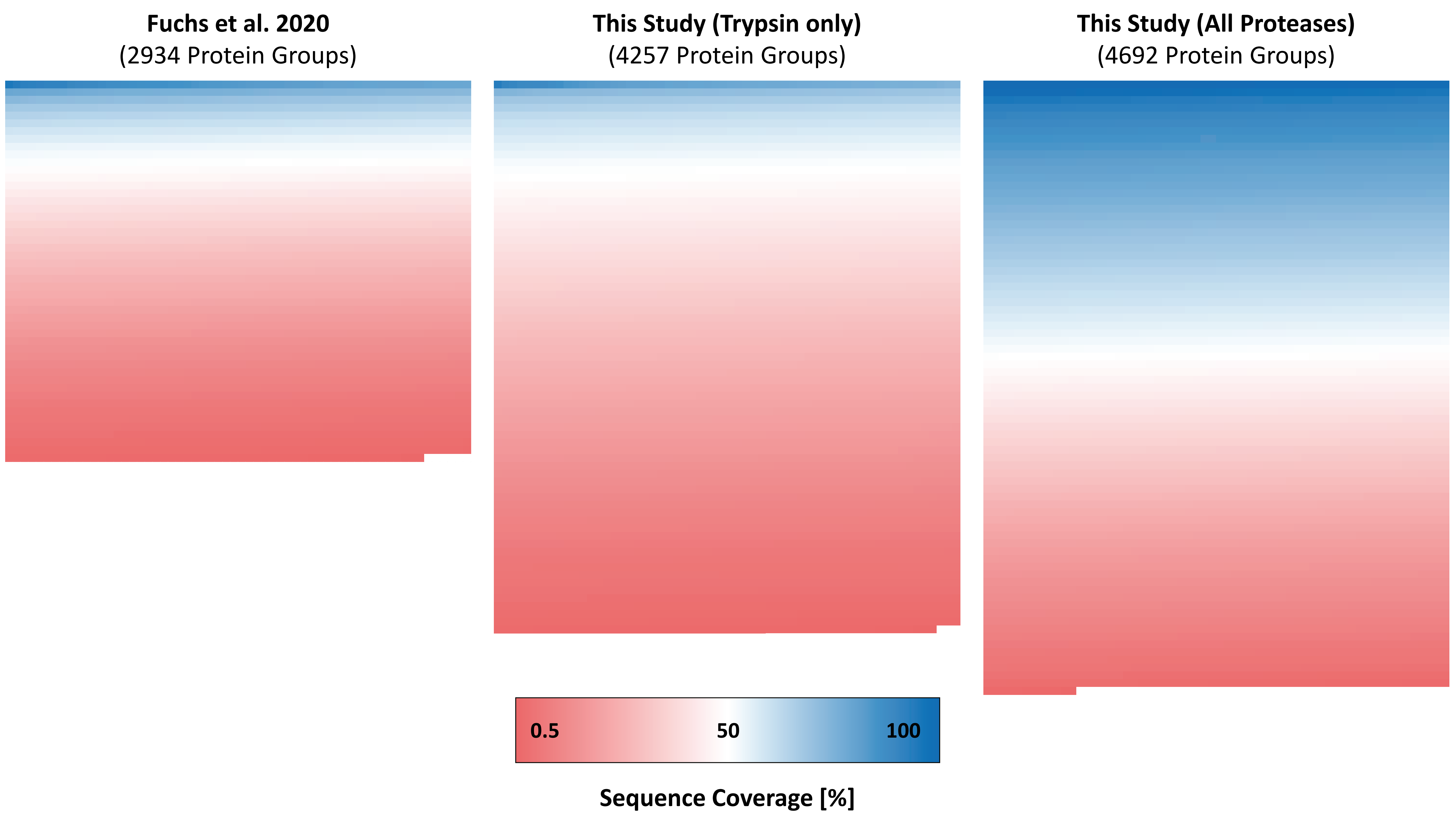

Supplemental Figure S2: Comparison of proteome coverage of the current study and the study presented by Fuchs et al. (2020). The area of the three elements corresponds to the number of identified proteins. Identified proteins were sorted and coloured in all three datasets based on the achieved sequence coverage (from blue = 100% to red = 0.5%), respectively.

Supplemental Figure S3: Representative MS/MS spectra of peptides specific for RNA editing site atp4eU416TIp98. Black arrows indicate amino acid positions affected by RNA editing. The Y-axis shows the relative intensity of the peaks to each other. The X-axis indicates the mass-to-charge (m/z) ratio of each peak. **A:** edited peptide. **B:** non-edited peptide. TOF: time-of-flight. CID: collision-induced dissociation. The spectra can also be viewed in detail online via MS-Viewer. Please refer to the Data Availability Statement.

A

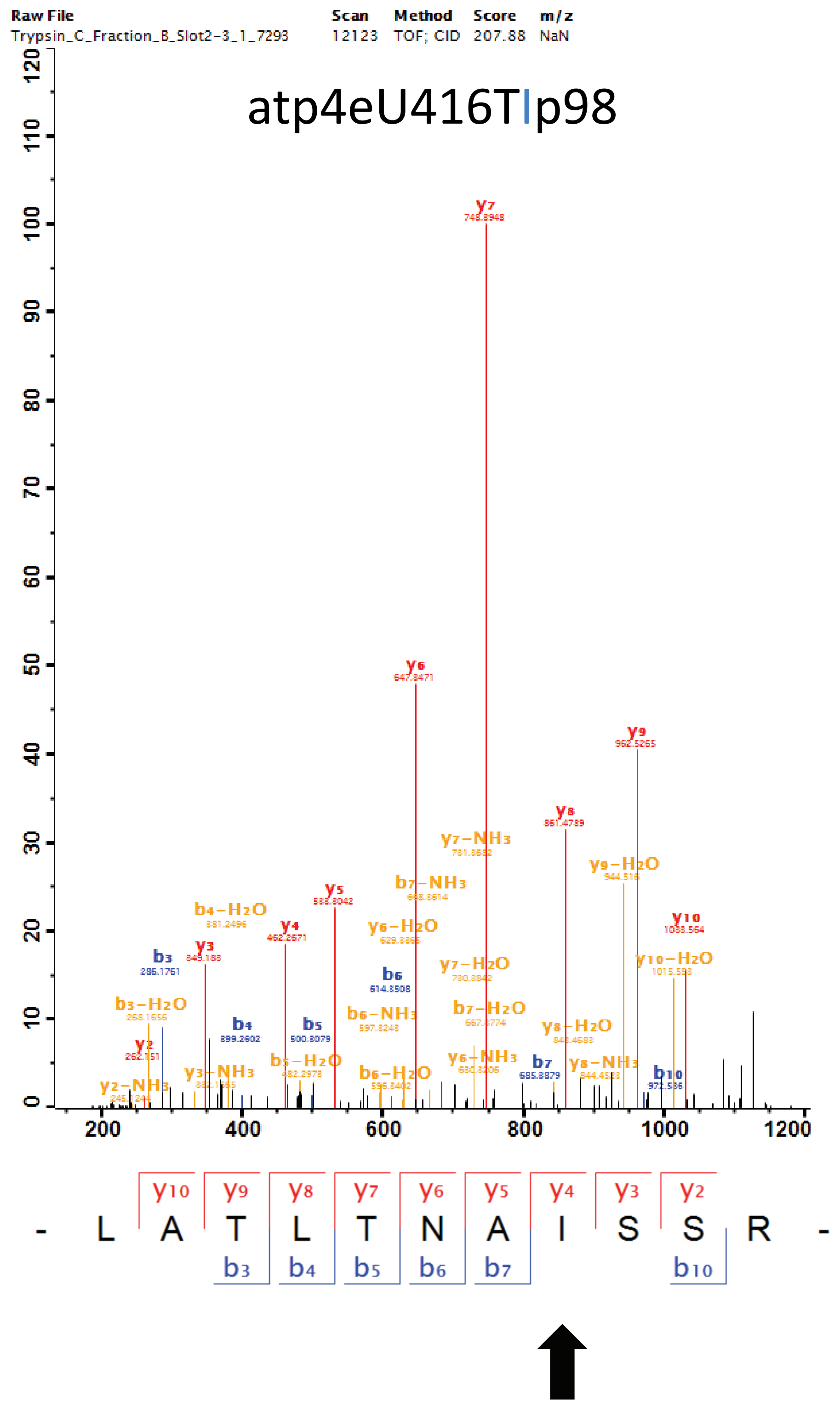

B

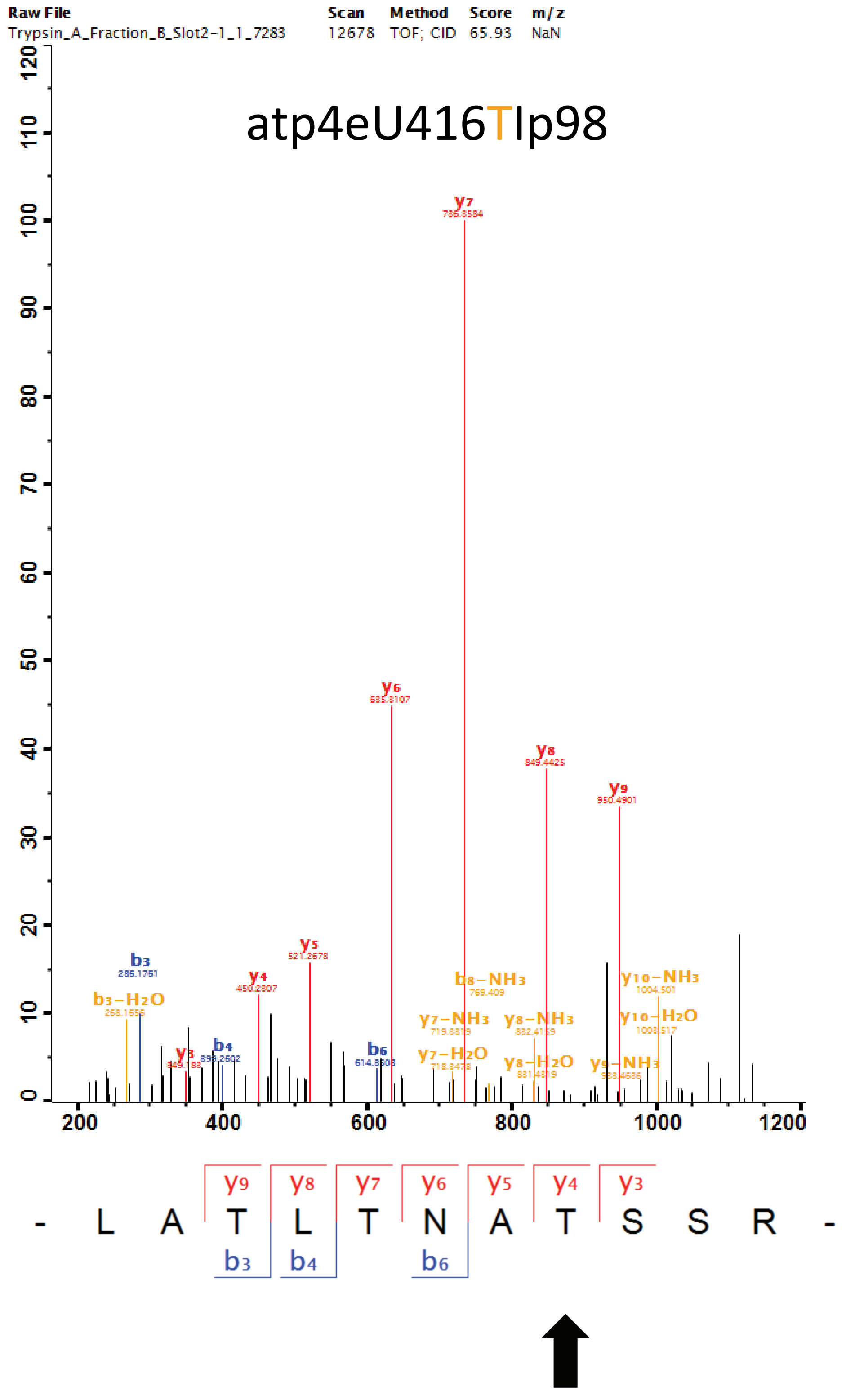

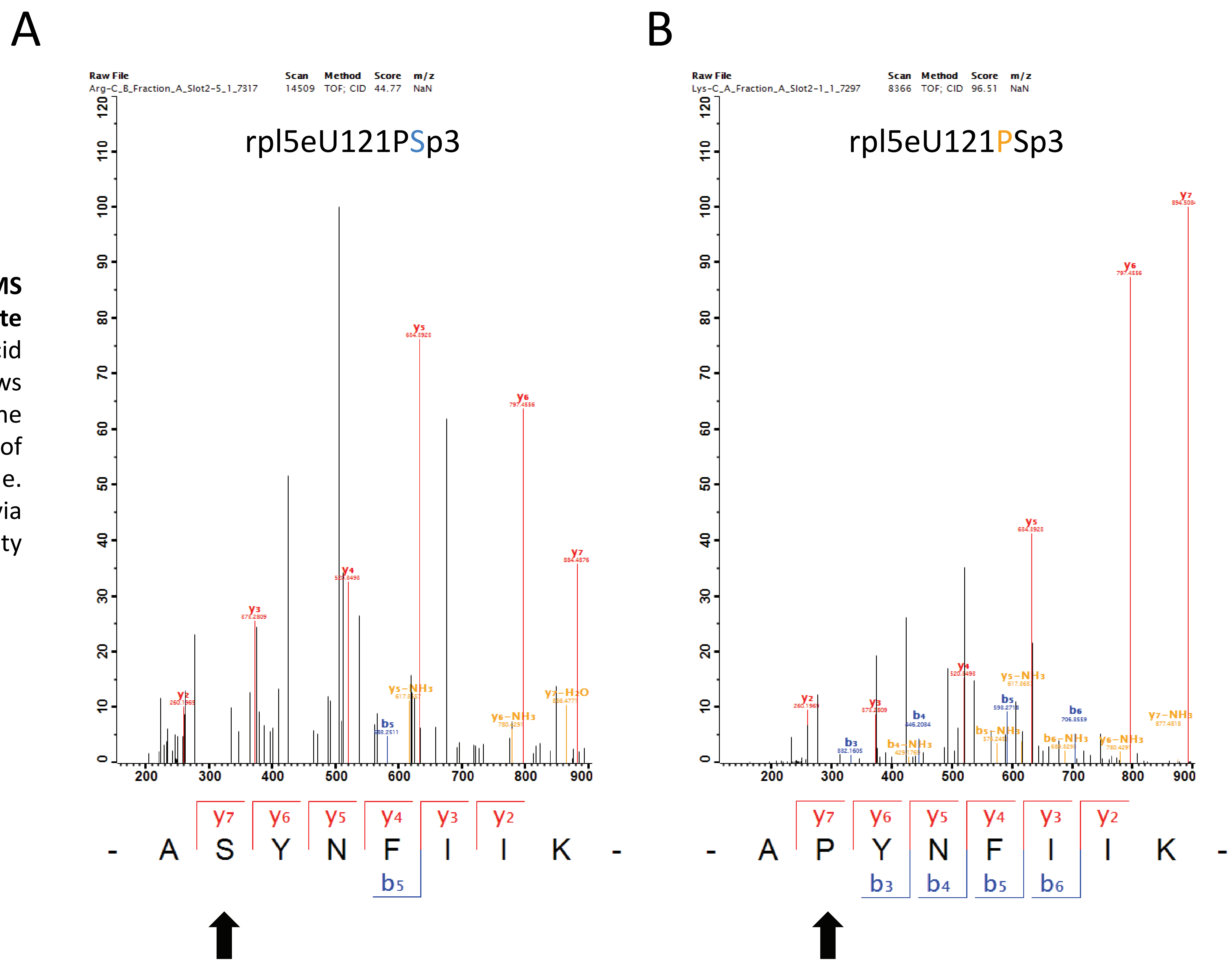

Supplemental Figure S4: Representative MS/MS spectra of peptides specific for RNA editing site rpl5eU121PSp3. Black arrows indicate amino acid positions affected by RNA editing. The Y-axis shows the relative intensity of the peaks to each other. The X-axis indicates the mass-to-charge (m/z) ratio of each peak. **A:** edited peptide. **B:** non-edited peptide. The spectra can also be viewed in detail online via MS-Viewer. Please refer to the Data Availability Statement.

Supplemental Figure S5: Representative MS/MS spectrum of a peptide specific for RNA editing sites rps3eU1571AVp97, rps3eU1580SFp11 and rps3eU1598SLp97. Black arrows indicate amino acid positions affected by RNA editing. The Y-axis shows the relative intensity of the peaks to each other. The X-axis indicates the mass-to-charge (m/z) ratio of each peak. The spectra can also be viewed in detail online via MS-Viewer. Please refer to the Data Availability Statement.

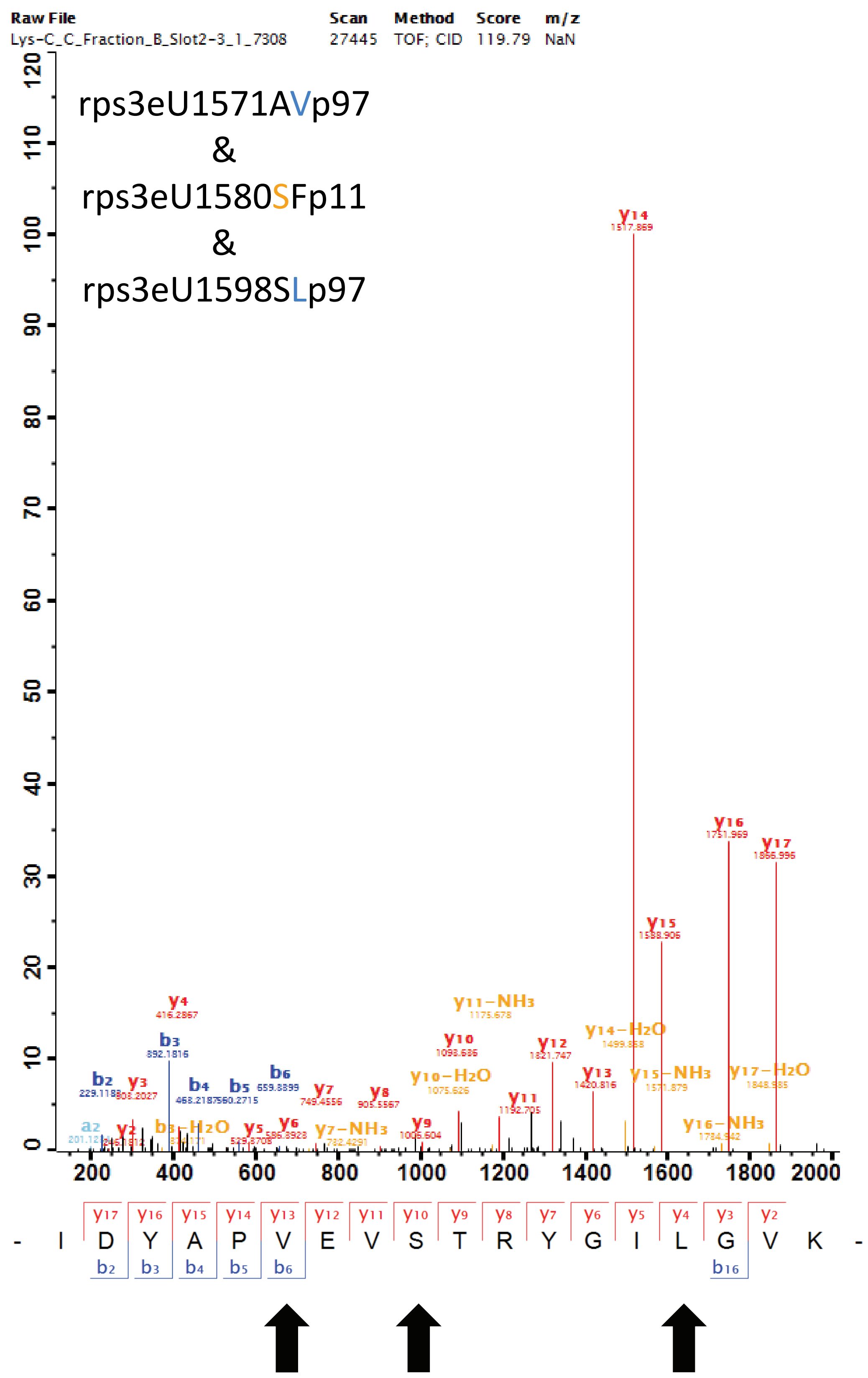

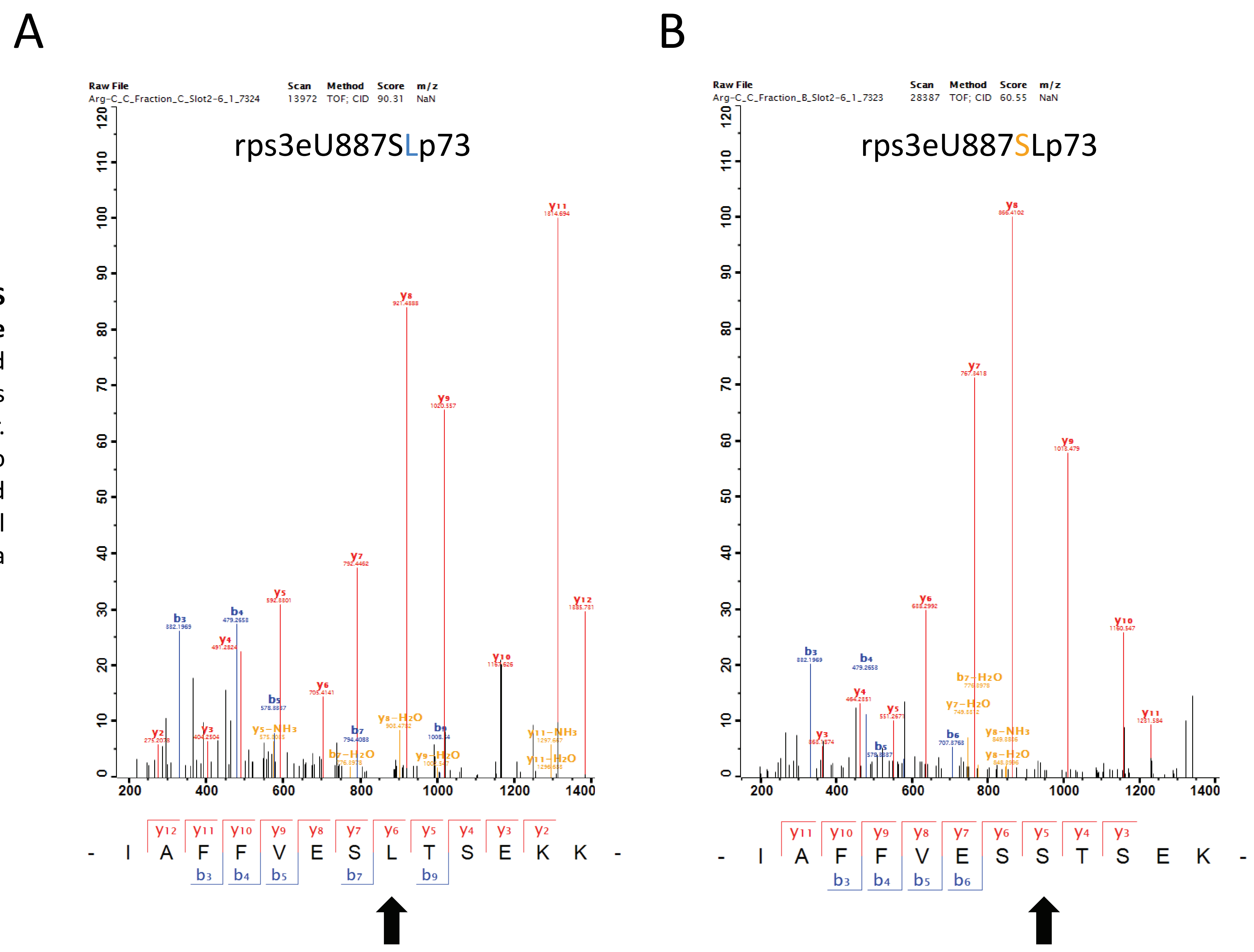

Supplemental Figure S6: Representative MS/MS spectra of peptides specific for RNA editing site rps3eU887SLp73. Black arrows indicate amino acid positions affected by RNA editing. The Y-axis shows the relative intensity of the peaks to each other. The X-axis indicates the mass-to-charge (m/z) ratio of each peak. **A:** edited peptide. **B:** non-edited peptide. The spectra can also be viewed in detail online via MS-Viewer. Please refer to the Data Availability Statement.

Supplemental Figure S7: Representative MS/MS spectra of peptides specific for RNA editing site atp4eU416TIp98.** Black arrows indicate amino acid positions affected by RNA editing. The Y-axis shows the relative intensity of the peaks to each other. The X-axis indicates the mass-to-charge (m/z) ratio of each peak. **A:** edited peptide. **B:** non-edited peptide. The spectra can also be viewed in detail online via MS-Viewer. Please refer to the Data Availability Statement.

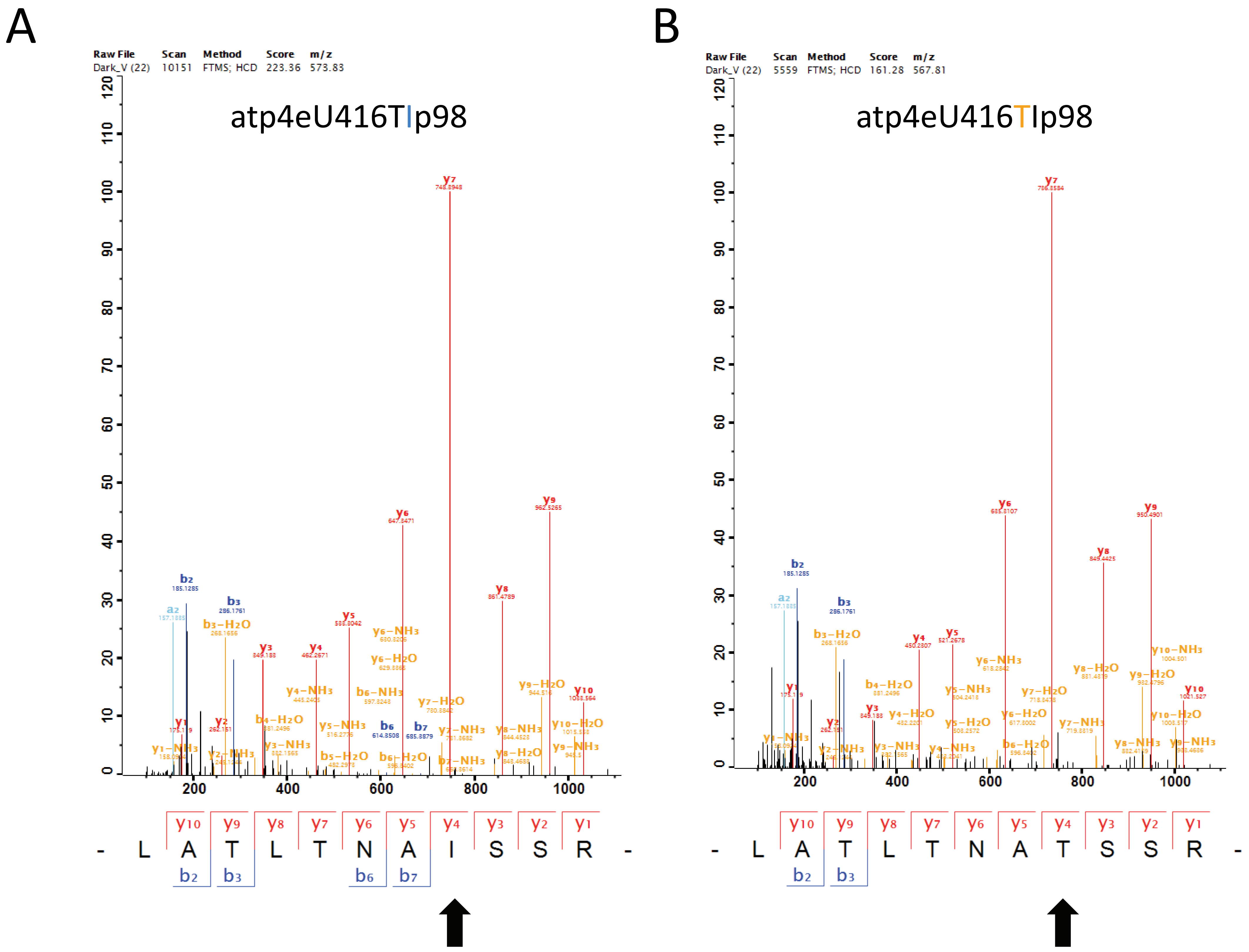

Supplemental Figure S8: Representative MS/MS spectra of peptides specific for RNA editing site rps3eU887SLp73. Black arrows indicate amino acid positions affected by RNA editing. The Y-axis shows the relative intensity of the peaks to each other. The X-axis indicates the mass-to-charge (m/z) ratio of each peak. **A:** edited peptide. **B:** non-edited peptide. The spectra can also be viewed in detail online via MS-Viewer. Please refer to the Data Availability Statement.

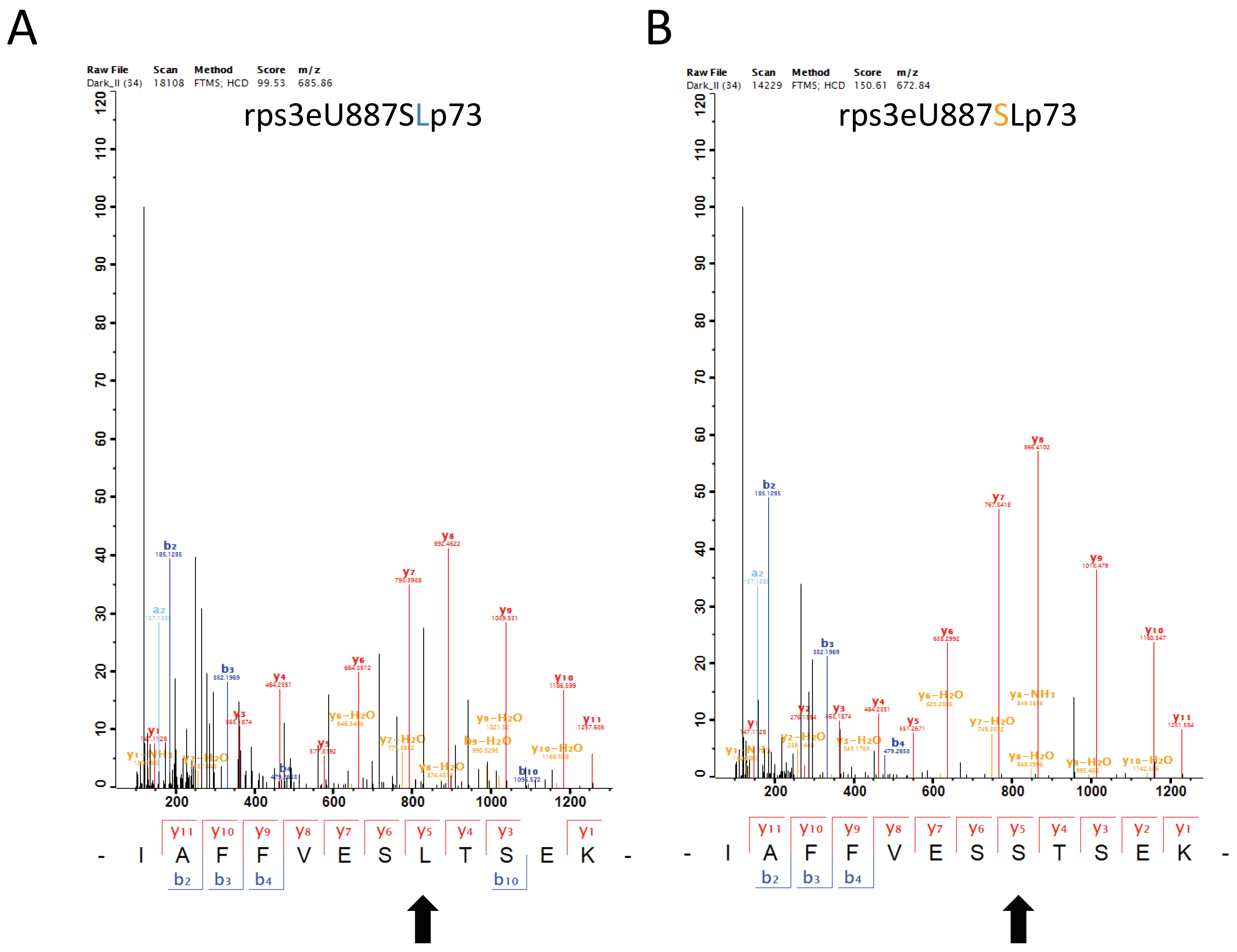

Supplemental Figure S9: Representative MS/MS spectra of peptides specific for RNA editing site rps3eU887SLp73. Black arrows indicate amino acid positions affected by RNA editing. The Y-axis shows the relative intensity of the peaks to each other. The X-axis indicates the mass-to-charge (m/z) ratio of each peak. **A:** edited peptide. **B:** non-edited peptide. The spectra can also be viewed in detail online via MS-Viewer. Please refer to the Data Availability Statement.

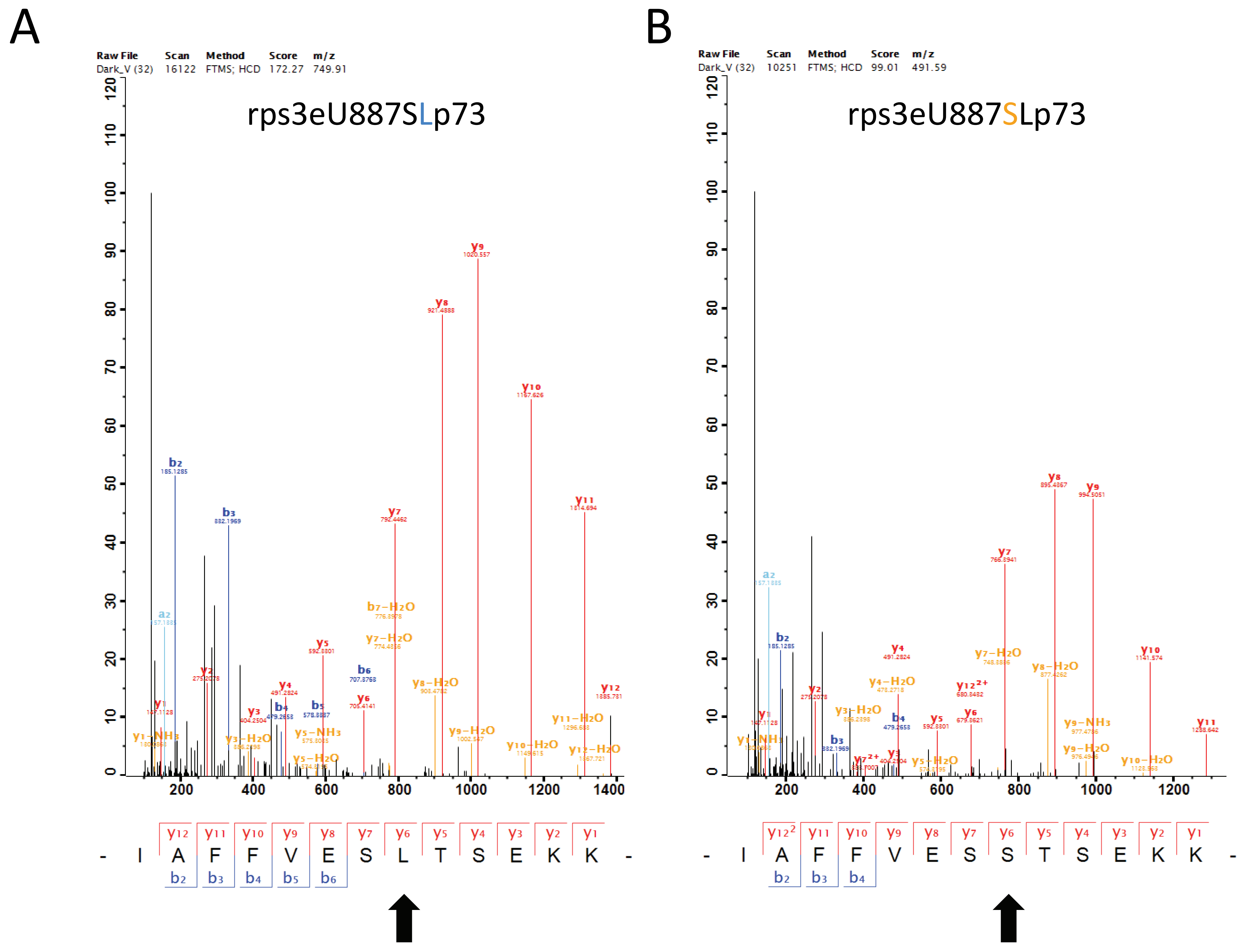

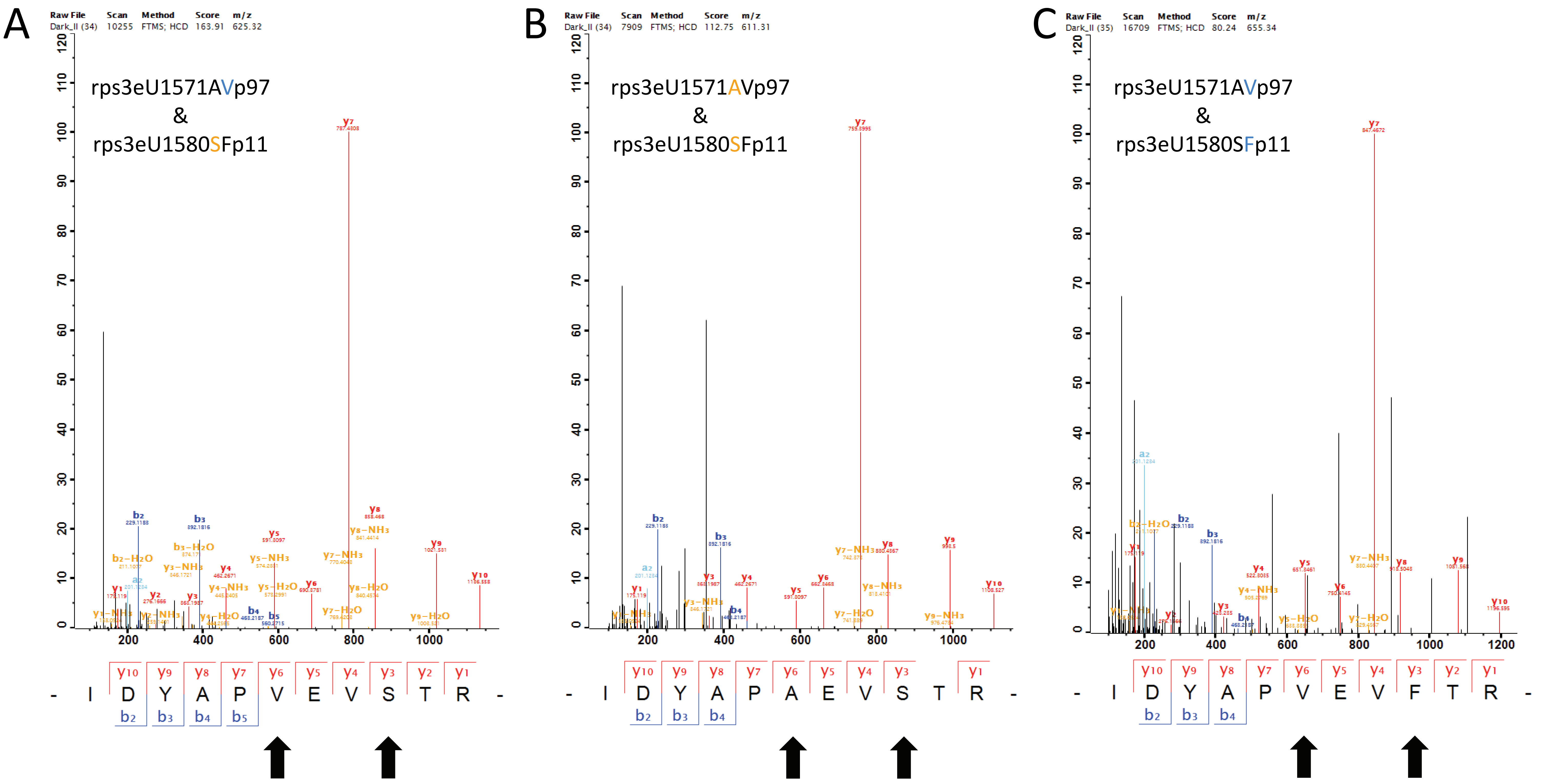

Supplemental Figure S10: Representative MS/MS spectra of peptides specific for RNA editing sites rps3eU1571AVp97 and rps3eU1580SFp11. Black arrows indicate amino acid positions affected by RNA editing. The Y-axis shows the relative intensity of the peaks to each other. The X-axis indicates the mass-to-charge (m/z) ratio of each peak. **A:** peptide edited at rps3eU1571AVp97 but not at rps3eU1580SFp11. **B:** peptide not edited at both sites. **C:** peptide edited at both sites. The spectra can also be viewed in detail online via MS-Viewer. Please refer to the Data Availability Statement.

Supplemental Figure S11: Representative MS/MS spectra of peptides indicative of alternative splicing events of AT3G20000 transcripts. The Y-axis shows the relative intensity of the peaks to each other. The X-axis indicates the mass-to-charge (m/z) ratio of each peak. **A:** tryptic peptide unique for the shorter TOM40 proteoform (AT3G20000.2). **B:** tryptic peptide unique for the longer TOM40 proteoform (AT3G20000.1). The spectra can also be viewed in detail online via MS-Viewer. Please refer to the Data Availability Statement.

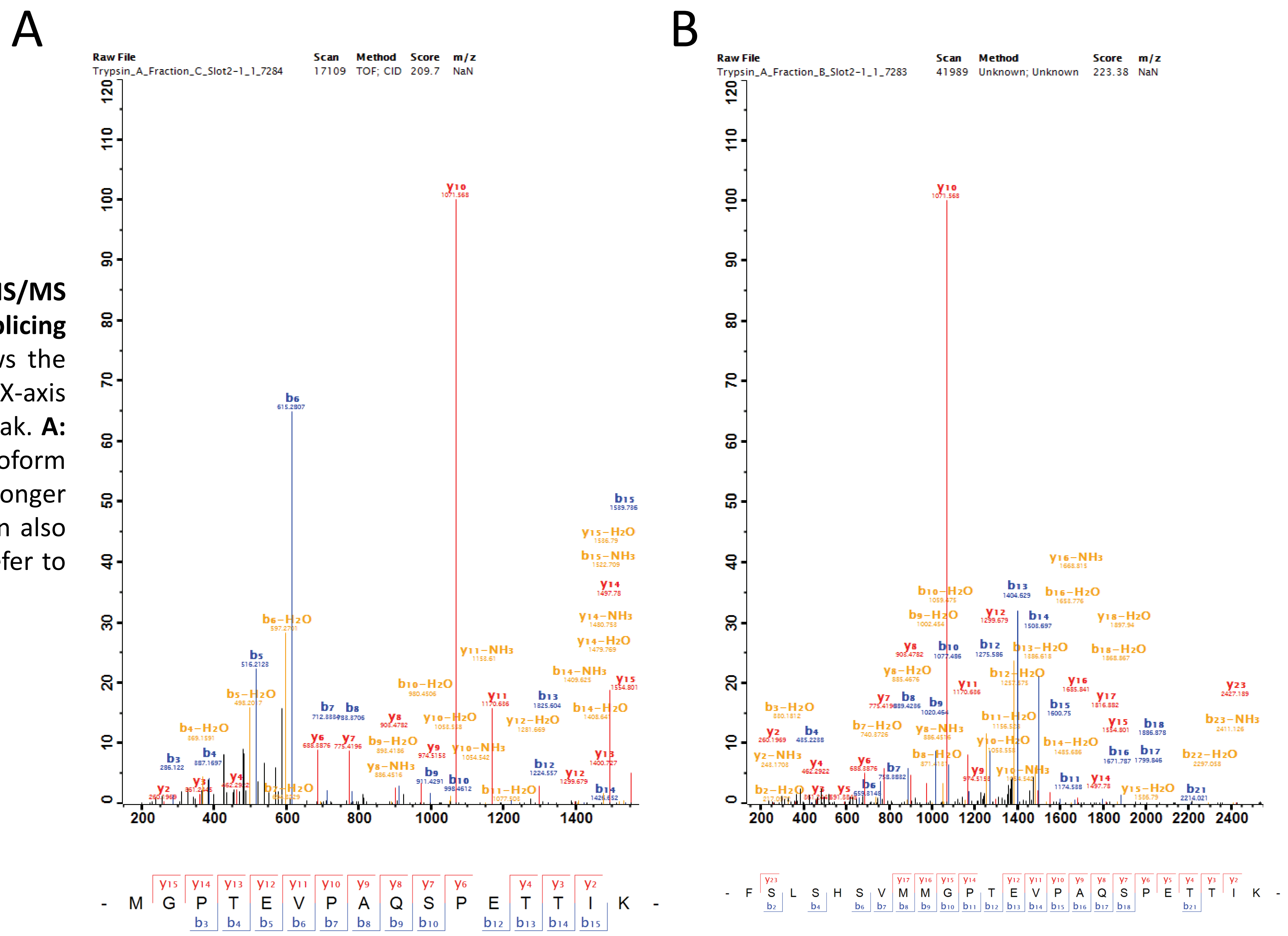

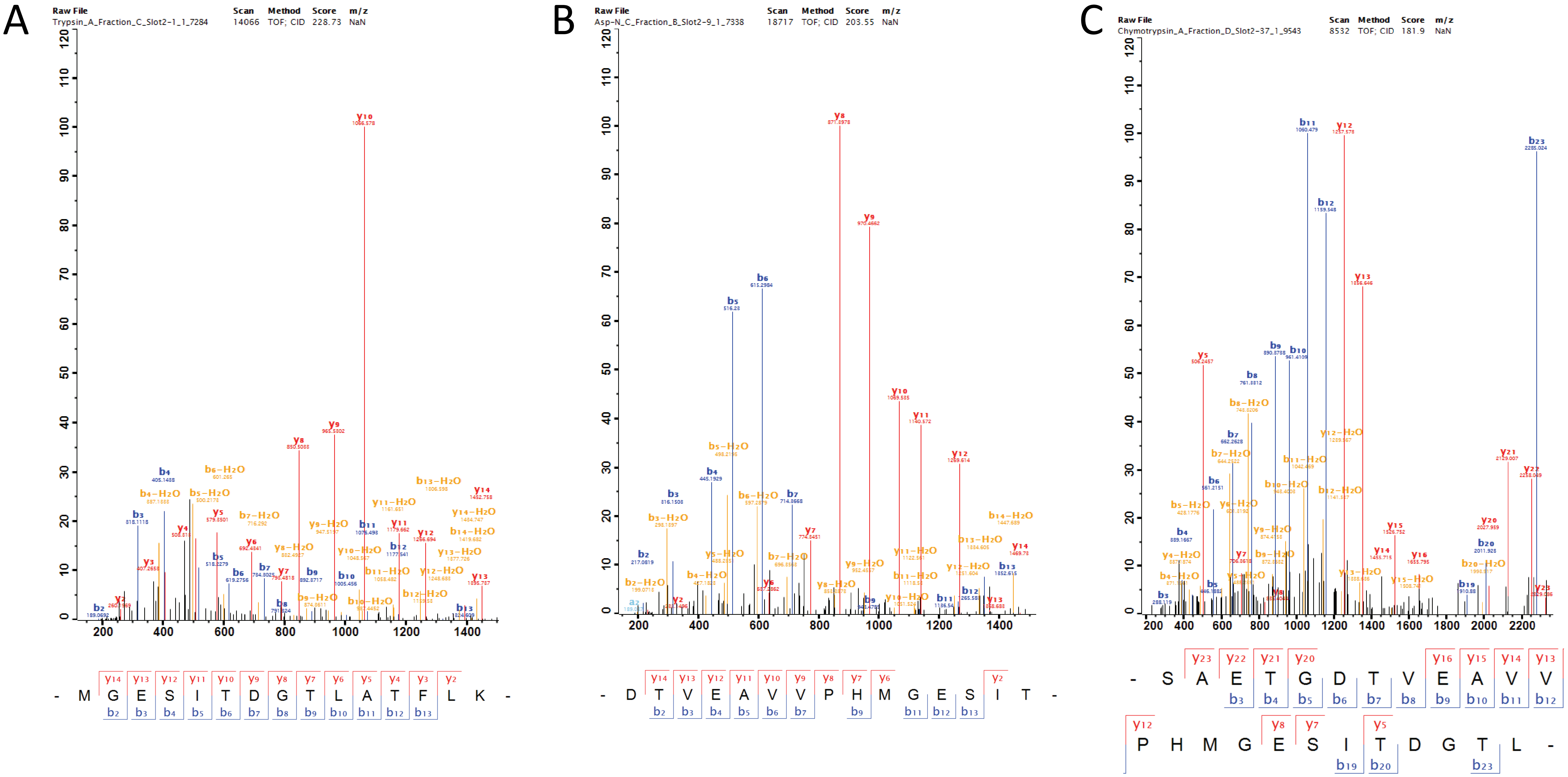

Supplemental Figure S12: Representative MS/MS spectra of peptides indicative of alternative splicing events of AT4G26910 transcripts. The Y-axis shows the relative intensity of the peaks to each other. The X-axis indicates the mass-to-charge (m/z) ratio of each peak. **A:** tryptic peptide unique for the shorter E2-OGDC proteoform (AT4G26910.3). **B:** peptide unique for the longer E2-OGDC proteoform (AT4G26910.1). **C:** peptide unique for the longer E2-OGDC proteoform (AT4G26910.1). The spectra can also be viewed in detail online via MS-Viewer. Please refer to the Data Availability Statement.
